# Supplementary material for: Surface triggered stabilization of metastable charge-ordered phase in SrTiO3
Source: Nat Commun. 2024 Feb 8;15:1180. doi: 10.1038/s41467-024-45342-8 (PMC10853244; doi:10.1038/s41467-024-45342-8)
Supplement: Supplementary file 1 — Supplementary Information [file 41467_2024_45342_MOESM1_ESM.pdf]

Supplementary Information for
Surface triggered stabilization of metastable charge-ordered phase in $SrTiO_3$

Kitae Eom[1,2†], Bongwook Chung[1†], Sehoon Oh[1], Hua Zhou[3], Jinsol Seo[4], Sang Ho Oh[4], Jinhyuk Jang[5], Si-Young Choi[5], Minsu Choi[1], Ilwan Seo[6], Yun Sang Lee[6], Youngmin Kim[7], Hyungwoo Lee[7,8], Jung-Woo Lee[9], Kyoungjun Lee[10], Mark Rzchowski[11], Chang-Beom Eom[10] ✉ & Jaichan Lee[1] ✉

[1]School of Advanced Materials Science and Engineering, Sungkyunkwan University (SKKU), Suwon, 16419, Republic of Korea.

[2]Department of Electronic Engineering, Gachon University, Seongnam, 13120, Republic of Korea.

[3]X-ray Science Division, Advanced Photon Source, Argonne National Laboratory, Lemont, IL 60439, USA.

[4]Department of Energy Engineering, KENTECH Institute for Energy Materials and Devices, Korea Institute of Energy Technology (KENTECH), Naju, 58330, Republic of Korea.

[5]Department of Materials Science and Engineering, Pohang University of Science and Technology (POSTECH), Pohang, Gyeongbuk 37673, Republic of Korea.

[6]Department of Physics, Soongsil University, Seoul, 06978, Republic of Korea.

[7]Department of Energy Systems Research, Ajou University, Suwon 16499, Republic of Korea

[8]Department of Physics, Ajou University, Suwon, 16499, Republic of Korea

[9]Department of Materials Science Engineering, Hongik University, Sejong 30016, Republic of Korea

[10]Department of Materials Science and Engineering, University of Wisconsin-Madison, Madison, WI 53706, USA.

[11]Department of Physics, University of Wisconsin, Madison, WI 53706, USA.

[†]These authors contributed equally: Kitae Eom, Bongwook Chung.

✉ e-mail: ceom@wisc.edu; jclee@skku.edu

This PDF file includes:

Supplementary Note 1: DFT calculation and symmetry analysis of the bulk LSTO

Supplementary Note 2: Film growth

Supplementary Note 3: Transport properties of LSTO films

Supplementary Note 4: Effect of the surface distortion on the transport properties

Supplementary Note 5: Optical conductivity measurements

Supplementary Note 6: Coherent Bragg rod analysis

Supplementary Note 7: STEM and EELS analysis

Supplementary Note 8: DFT calculation and symmetry analysis of the LSTO/STO heterogeneous slab

Supplementary Note 9: Verification of surface structure

Supplementary Note 10: Effect of oxygen adsorption on the film surface for electrical properties

Supplementary Note 11: Attenuation of the surface charge-ordered phase

Supplementary Note 12: Charge-ordered phase in different La-doping concentration

Supplementary Note 1: DFT calculation and symmetry analysis of the bulk LSTO

We investigate the atomic and electronic structures of the bulk $La_{0.25}Sr_{0.75}TiO_3$ (LSTO) using the density functional theory (DFT) with semi-empirical Hubbard U correction. Considering the experimentally observed oxygen octahedral rotation pattern[1,2], we assume bulk LSTO has $a^0a^0c^-$ Glazer`s notation. We optimize the lattice constants and the internal atomic coordination of metallic bulk LSTO based on the $2a \times 2a \times 2c$ cell. The optimized in-plane and out-of-plane lattice constants with the anti-phase octahedral rotation distortion ($a^0a^0c^-$ in Glazer's notation) are a = 0.39679 and c = 0.40159 nm, respectively. For the La_{Sr} doping configuration, we investigate several doping configurations, and the total energy difference between different doping configurations is up to 3 meV/formula unit. A configuration where La_{Sr} dopants are linearly aligned along the out-of-plane direction is found to be energetically favorable, and this doping configuration is adopted for all the calculations.

It was theoretically shown that breathing oxygen octahedral distortion, which expands or contracts the oxygen octahedra nearest neighbor unit cells, allows charge ordering (CO) in half-doped titanate double perovskite[3]. Therefore, we initially introduce periodic modulation of oxygen octahedral distortion that consists of expansion and contraction of TiO_6 octahedra and obtained the charge-ordered LSTO bulk structure. It shows the antiferromagnetic Mott insulating behavior with the localized Ti 3d band as shown in Supplementary Figure 1. The optimized lattice constants of this structure obtained from the $2a \times 2b \times 4c$ cell (Supplementary Fig. 2a) are a = 0.39869, b = 0.39506, and c = 0.40371 nm, respectively.

We analyze the lattice modulation of the CO state bulk LSTO. The lattice modulations of the CO state bulk LSTO are decomposed into distortion modes, i.e., $\Delta r = \sum_i c_i \Phi_i$, where Δr is the lattice modulation vector of the bulk LSTO as the atomic displacement vector from the symmetrical structure (Supplementary Fig. 2b), c_i is the coefficients of the normalized distortion mode Φ_i (i = 1, 2, 3, z- and rem), respectively. For the distortion mode vector Φ_i (i =

1, 2, 3, z- and rem), Φ_1 is the inter-layer breathing mode that is oxygen octahedra volume expansion and contraction along the **z** direction (Fig. 1c). Φ_2 is the intra-layer breathing mode that an in-plane nearest-neighbor oxygen octahedron to either expand or contract. This expansion and contraction of oxygen octahedra along the in-plane direction have a one-layer interval and out-of-phase motions along the *z* direction (Fig. 1d). Φ_3 is the Jahn-Teller type distortion mode which has different Ti-O bond lengths along the **x** and **y** directions. Similar to Φ_2, Φ_3 also has a one-layer interval and out-of-phase motions along the **z** direction (Fig. 1e). Φ_{z-} is the antiferrodistortive rotation ($a^0a^0c^-$ in Glazer's notation) i.e., an anti-phase octahedral rotation along the **z** direction (Fig. 1f). Note that these distortion mode vectors Φ_i (i = 1, 2, 3, z-), are orthogonal to each other. The remaining term (Φ_{rem}) mainly comes from the displacements of atoms near the La_{Sr} dopants (Supplementary Fig. 3). d_is are oxygen displacement with respect to the high symmetric structure, which is defined as the multiply c_i by individual oxygen displacement of the normalized distortion mode vector Φ_i (i = 1, 2, 3, z-). Oxygen octahedral rotation angle θ_{z-} of antiferrodistortive rotation is converted from d_{z-}. The obtained coefficients c_is of the CO phase are $c_1 = 0.170$, $c_2 = 0.193$, $c_3 = 0.104$, $c_{z-} = 1.79$, and $c_{rem} = 0.369$. The oxygen displacements and the oxygen octahedral rotation angle of the CO phase are $d_1 = 4.24$ pm, $d_2 = 3.41$ pm, $d_3 = 2.59$ pm, and $\theta_{z-} = 9.08°$, respectively.

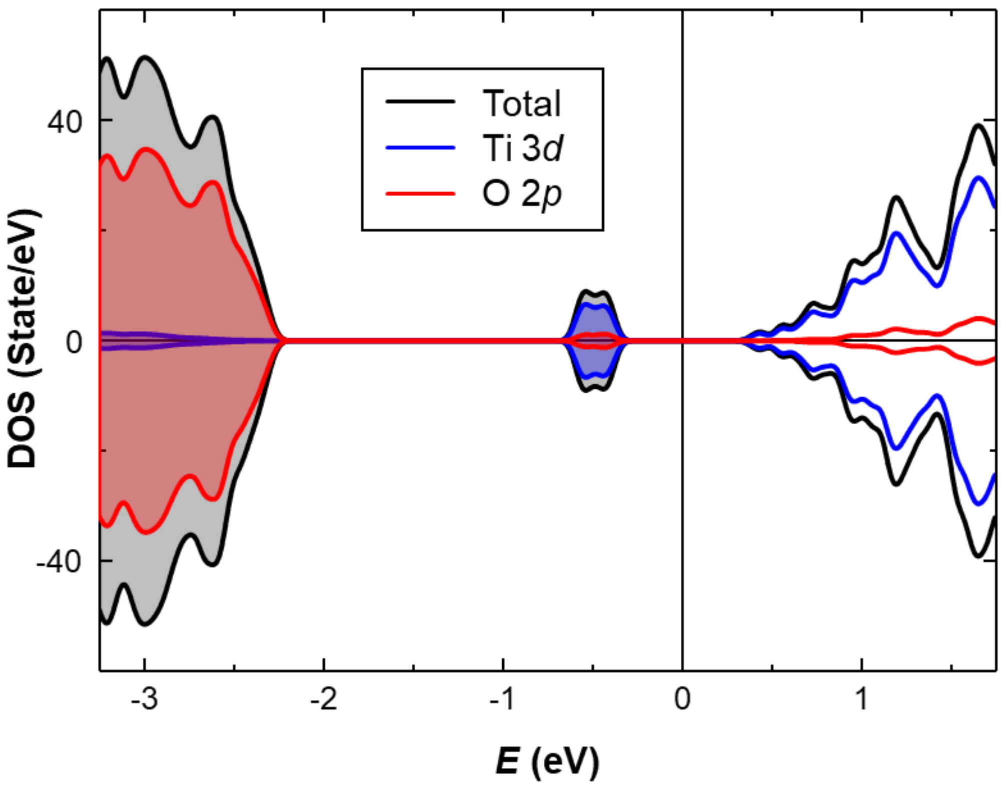

Supplementary Fig. 1 | Spin-polarized DOS of the charge-ordered insulating bulk LSTO. Total DOS is represented by black lines, and those projected onto the Ti 3*d* and O 2*p* orbitals are represented by blue and red lines, respectively. Positive and negative regions correspond to the up-spin and the down-spin states, respectively. The midpoint between the energies of the lowest unoccupied state and the highest occupied state is set to be zero and represented by the vertical line.

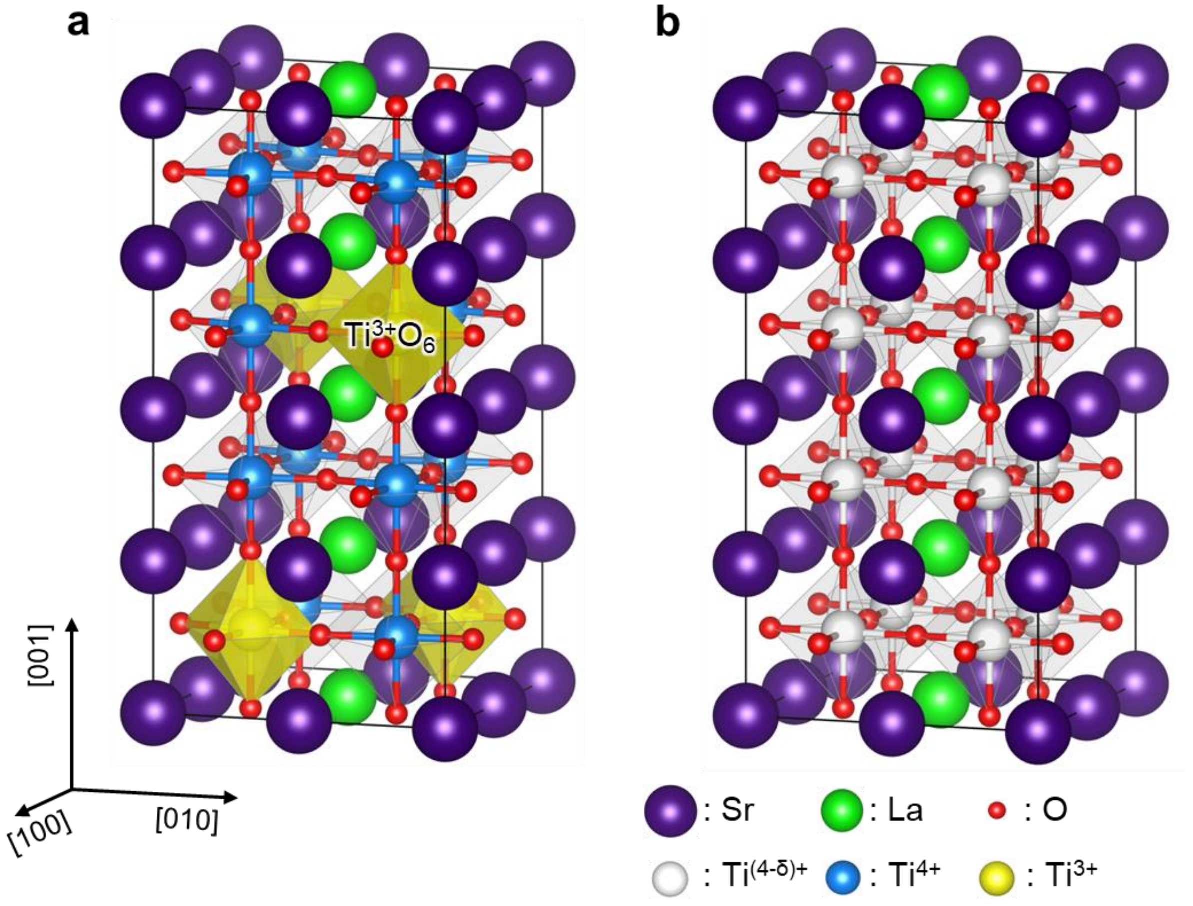

Supplementary Fig. 2 | Atomic structure of the LSTO bulk. a Charge-ordered metastable structure of the LSTO bulk. The obtained structure shows the periodic expansion and contraction of the oxygen octahedral pattern. **b** Symmetric structure of the LSTO bulk without periodic structural distortion. In **a**, **b** the linear configuration of the La_{Sr} dopant in the $2a \times 2b \times 4c$ cell is adopted for both structures. The unit cell is represented by black lines. Purple, chartreuse, red, blue, yellow, and white spheres represent the Sr, La, and O atoms and Ti^{4+}, Ti^{3+}, and partially filled $Ti^{(4-δ)+}$ cations, respectively. In **a** the yellow shades on the $Ti^{3+}O_6$ octahedral surface are guides for eyes.

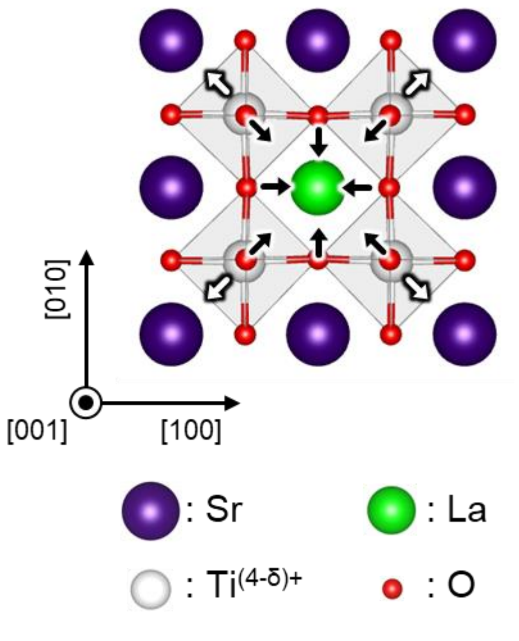

Supplementary Fig. 3 | Schematics of the remaining term Φ_{rem} for the LSTO bulk. Φ_{rem} mainly consists of the displacements near La_{Sr}. Purple, chartreuse, red, and white spheres represent the Sr, La, and O atoms and $Ti^{(4-\delta)+}$ cation, respectively.

Supplementary Note 2: Film growth

Digitally controlled $La_{0.25}Sr_{0.75}TiO_3$ (LSTO) films were grown on (001)-oriented $SrTiO_3$ by pulsed laser deposition in an ambient oxygen/ozone mixture with a pressure of 10^{-5} Torr and substrate temperature of 650 °C. The RHEED intensity oscillations obtained from specular spots exhibit layer-by-layer growth as shown in Supplementary Figure 4a. All the surfaces, regardless of thickness, are atomically flat and smooth (Supplementary Fig. 4b). The film thicknesses ranged from 4 to 20 unit cells (u.c.) and were controlled by monitoring the RHEED oscillations during the growth (Supplementary Fig. 4a).

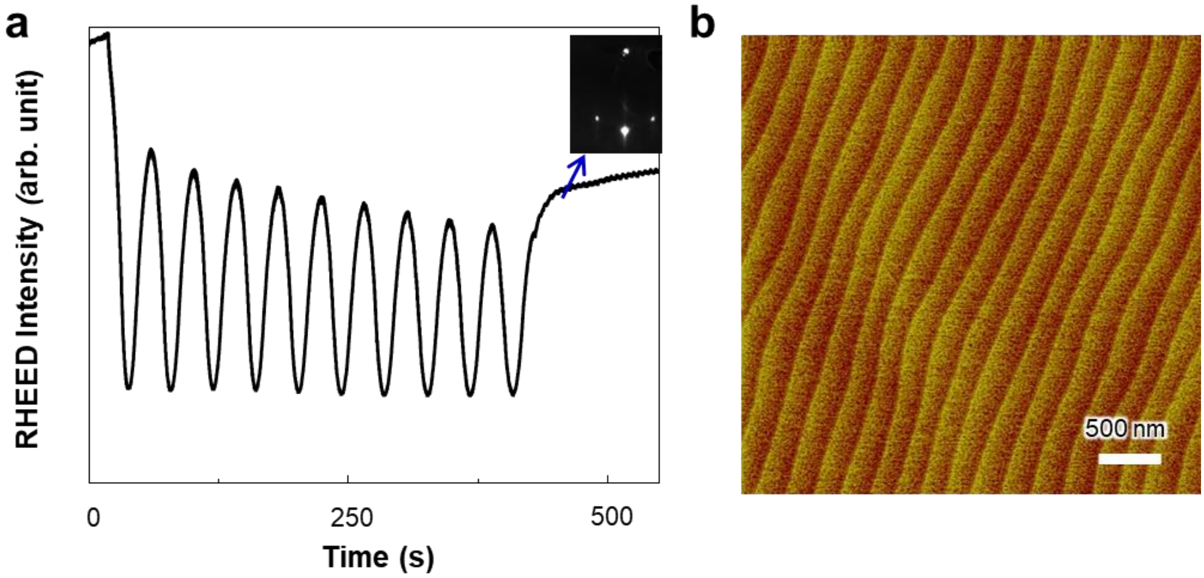

Supplementary Fig. 4 | Epitaxial growth of LSTO films. a RHEED intensity oscillations during growth of $La_{0.25}Sr_{0.75}TiO_3$ film grown on $SrTiO_3$ substrate. (Inset) Typical RHEED patterns of the LSTO film. **b** AFM image of the LSTO film which shows the atomically smooth film surface.

Supplementary Note 3: Transport properties of LSTO films

Temperature-dependent resistivity of 6 u.c. thick LSTO film exhibits a semiconducting behavior. The resistivity-temperature curve of 6 u.c. thick LSTO film closely follows a thermally activated adiabatic small polaron hopping[4-7] (Supplementary Fig. 5). In this case, the electron hops between Ti^{3+} sites and the next neighbor of Ti^{4+} sites. The temperature-dependent resistivity for the small polaron hopping (SPH) model in the adiabatic regime can be expressed as:

$$\rho(T) = \rho_0 T \exp(E_A/k_B T) \quad (1)$$

where ρ_0 is a pre-exponential factor, T is the absolute temperature, k_B is the Boltzmann constant, E_A is the activation energy. E_A is estimated to be 103.39 meV.

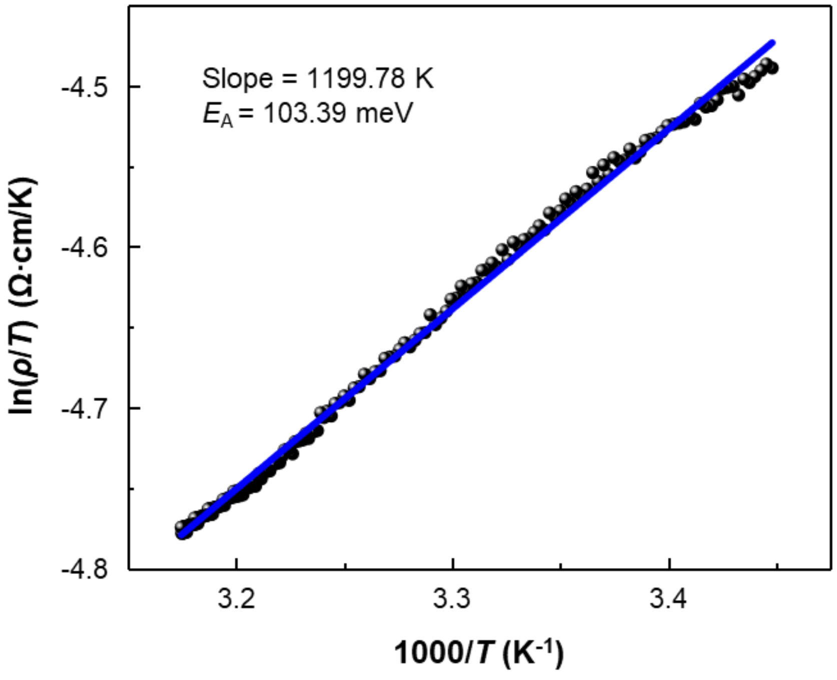

Supplementary Fig. 5 | Temperature versus resistivity curve of 6 u.c. thick of LSTO film plotted as the adiabatic small polaron hopping model. The blue line shows linear fitting curves to estimate activation energy (E_A).

Supplementary Note 4: Effect of the surface distortion on the transport properties

To verify the influence of the surface distortion, we grew a 10 u.c. thick STO capping layer on top of various thicknesses of the LSTO thin films and found its significant effects on the transport properties. We measured the room temperature resistivity of the samples with and without the STO capping layer (Supplementary Fig. 6a). For thinner LSTO films, the resistivity significantly decreased with the growth of the STO capping layer on the top. This effect was less pronounced for thicker films. Particularly, 6 u.c. and 7 u.c. thick LSTO films exhibited an insulating behavior without capping layer but demonstrated metallic behavior with the capping layer of 10 u.c. thickness (Supplementary Fig. 6b). These findings suggest that the insulating ground state in ultrathin LSTO films originates from the top surface, as described in the main text, rather than from the bottom interface or the bulk interior of the film.

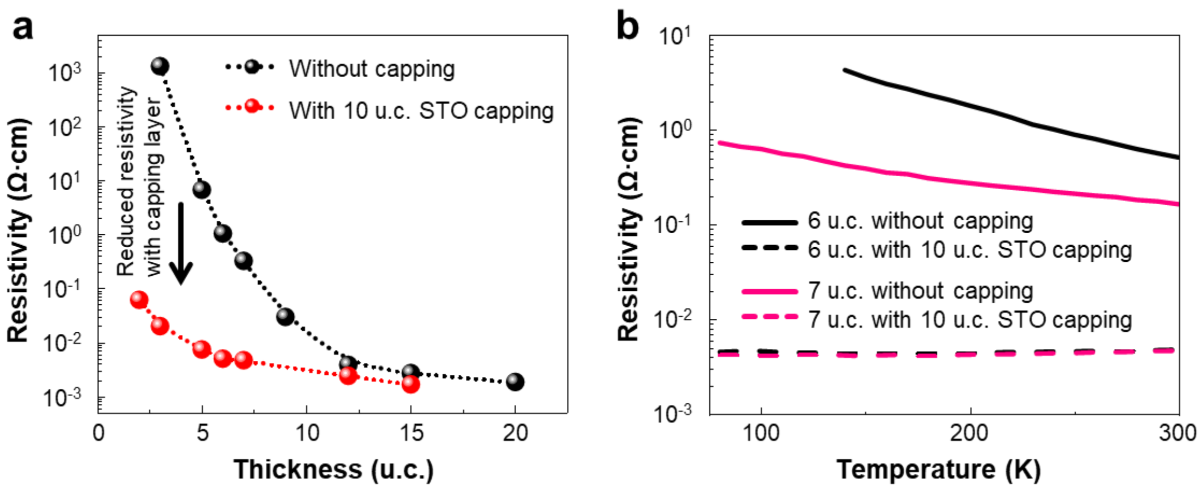

Supplementary Fig. 6 | Effect of STO encapsulation on transport properties of the LSTO films. a Thickness-dependent resistivity of LSTO films with and without 10 u.c. thick STO capping layer measured at room temperature. **b** Resistivity as a function of temperature for 6 u.c. and 7 u.c. thick of LSTO films with and without 10 u.c. thick of STO capping layer, respectively.

Supplementary Note 5: Optical conductivity measurements

To obtain the complex optical conductivity spectra $\tilde{\sigma}(\omega)[\equiv \sigma_1(\omega) + i\sigma_2(\omega)]$, we performed ellipsometry at room temperature which directly yields the complex optical constants without Kramers-Kronig analysis. We measured the ellipsometric angular spectra of the superlattices using a spectroscopic ellipsometer (VASE, J. A. Woollan). The angles of the incident light and the spectral ranges were 65°, 70°, and 75° (angle between the plane of the samples and the incident beam), and 0.7 – 5.5 eV, respectively. The complex dielectric functions, $\tilde{\varepsilon}(\omega)\left[\equiv 1 + i\frac{4\pi}{\omega}\tilde{\sigma}(\omega)\right]$, were estimated with a three-phase model composed of one Drude component and two Lorentz oscillators. The two Lorentz oscillators, located at 1.5 eV and 3.4 eV, correspond to the incoherent mode and the charge transfer excitation, respectively. The dielectric function yielded from the three-phase model fitting was checked by point-by-point fitting. The two dielectric functions, extracted by different fitting methods, were consistent with each other. The optical data were also confirmed by comparing a reflectivity directly measured by the conventional FT-IR/grating-type spectrometers in a photon energy range of 0.1 eV – 5.5 eV. The reflectivity taken from the direct measurement was in accordance with the resistivity extracted from the ellipsometry.

The incoherent mode near 1.3 eV, observed in our LSTO films (Fig. 2e), was not present at the $SrTiO_3$ substrate (Supplementary Fig. 7). This implies that the incoherent spectral peaks are the intrinsic behavior of the LSTO films. In the high energy region $\sigma_1(\omega)$ of LSTO, similarly to the case of undoped STO, a strong absorption above 4 eV is observed due to the charge transfer excitation (CTE) from O $2p$ to Ti t_{2g} bands, as shown in Supplementary Figure 7. We note that the CTE edge of the LSTO spectra shifts to higher energies compared with that of an undoped STO single crystal, which has been observed for the doped STO bulk samples[8]. Notably, the La ion doping leads to the emergence of spectral weight below CTE.

To determine an optical gap, we extracted an onset energy (E_{Onset}) of optical conductivity spectra. Extrapolating the low energy shoulder of the incoherent mode peak near 1.3 eV of semiconducting 6 u.c. thick LSTO film (Supplementary Fig. 8), we obtained the onset energy (E_{Onset}) of 600 meV. In the small polaron system, the optical gap is regarded as twice the polaron binding energy (E_p) due to the two relaxation channels which can lead to the hopping transfer or on-site relaxation[5,7]. The activation energy (E_A) is related to the polaron binding energy (E_p) and electron transfer integral (J). The transfer integral (J) is determined by tunneling process, thus depends on the wavefunction overlap or orbital overlap between adjacent sites. In addition, intersite Coulomb repulsion (E_C) should be considered especially in highly doped systems since it disturbs electron hopping process [5,9]. The relationship of E_A, E_P, J, and E_C in the SPH model can be expressed as[5]:

$$E_A = 1/2E_P - J + E_C. \quad (2)$$

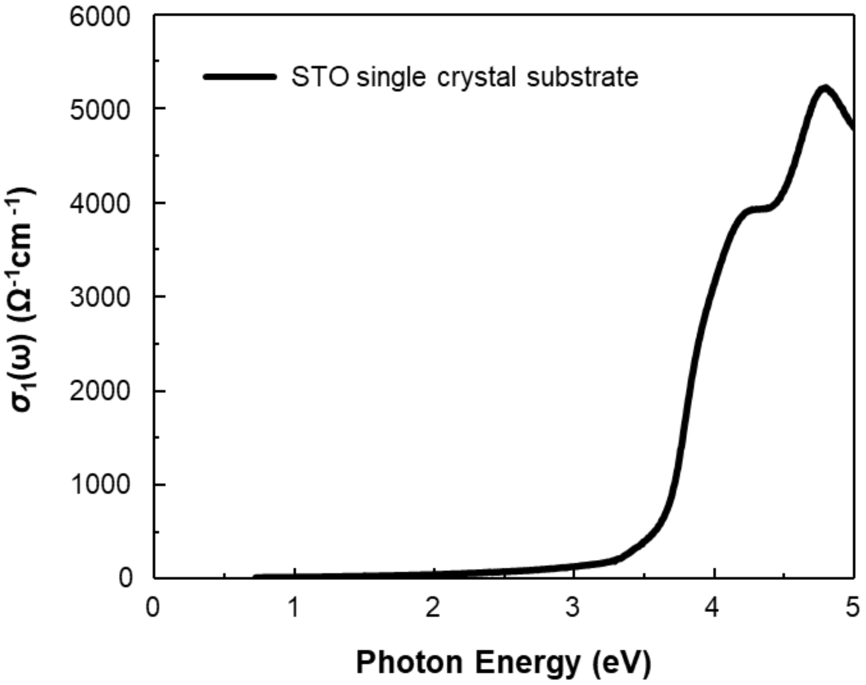

Supplementary Fig. 7 | Optical conductivity spectra of STO substrate. Optical conductivity is obtained from the ellipsometry of the STO substrate. Note that no incoherent part of the spectra is observed.

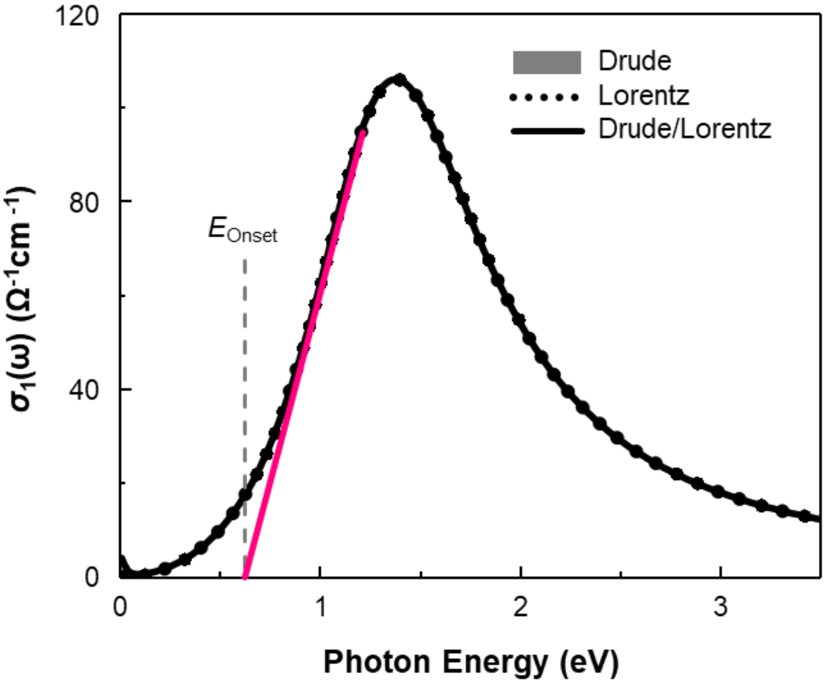

Supplementary Fig. 8 | Optical conductivity spectra of the 6 u.c. thick LSTO substrate. An onset energy (E_{Onset}) is determined from the linear extrapolation of the low energy shoulder (magenta line) of the peak near 1.3 eV.

Supplementary Note 6: Coherent Bragg rod analysis

Experimentally recorded CTR data were first background subtracted using the 2D detector images, and then properly corrected for geometric and polarization factors. The resulting structural factors were used for the subsequent COBRA analysis. The total 3D electron densities (EDs) for the complete atomic structures of the thin film system, including the epitaxial thin film unit cells and the top few unit cells of the substrate (e.g. typically 6-8 substrate unit cells), were reconstructed from the complete set of CTRs by using a Fourier phase retrieval iterative technique, known as coherent Bragg rods analysis (COBRA), through a self-developed MATLAB code universally optimized for systems with symmetry lower than 4 mm (or a simple four-fold symmetric system). Within each iteration in COBRA analysis, real space and reciprocal space constraints are alternatively applied to recover the phase information at each reciprocal space point from measured CTRs. In the cases where thin film and substrate symmetries are different (i.e., different octahedral rotation patterns and amplitudes), the epitaxial thin film may form different structural domains (as compared to the bulk substrate). In this case, the reconstructed thin film ED contains thin film structural information that is folded into the substrate-defined in-plane unit cell. Specific information such as oxygen octahedral rotation can be deduced from the broadening of the folded-unit cell ED profile induced by the rotation.

The general approach for obtaining experimental errors based on traditional fitting models is not applicable to COBRA-generated Eds. A method called "Noise Analysis", based on bootstrap-type statistical analysis was used to determine the uncertainties in COBRA results. This method applies additional random noises to the experimentally obtained CTR data and then analyzes the degree of scattering in the parameters extracted from EDs.

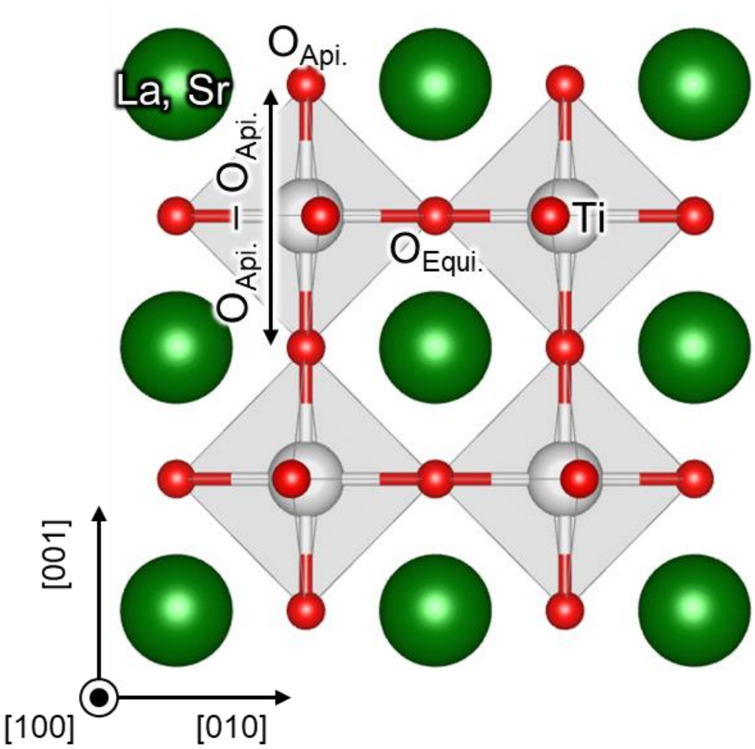

Supplementary Fig. 9 | Definition for the height of oxygen octahedron. The atomic structure of the LSTO is shown. Green, white, and red spheres represent (La, Sr), Ti, and O atoms, respectively. The apical oxygen-to-oxygen distance ($O_{Api.}$ – $O_{Api.}$) is defined as described above figure, which is used for quantitative analysis of the octahedral modulation in this study.

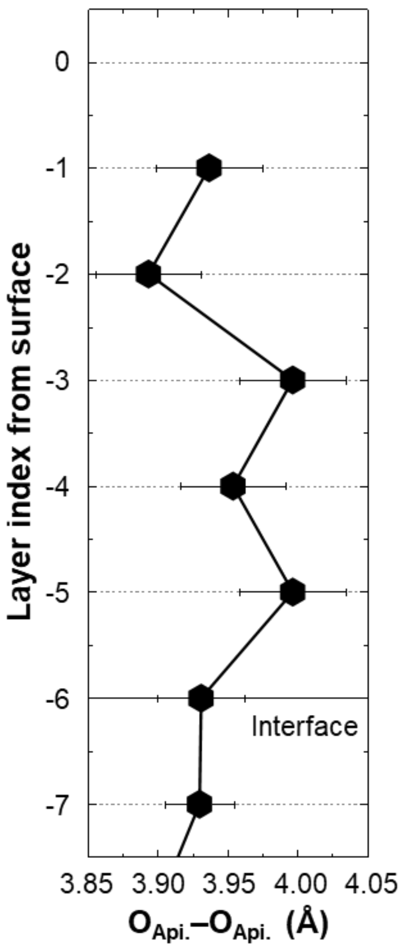

Supplementary Fig. 10 | COBRA result of LSTO 6 u.c. on STO. A layer resolved distance between apical oxygen atoms, which is defined as shown in Supplementary Fig. 9. The result is in good agreement with DFT calculation results (Fig. 4c).

Supplementary Note 7: STEM/ EELS analyses

EELS 2D scan was conducted across the LSTO/STO interface to trace the electron confinement at Ti 3*d* orbitals. When an additional electron is confined at the Ti 3*d* orbital, it tends to occupy the t_{2g} orbital which is at a lower energy state due to the crystal field splitting. If the t_{2g} orbital is occupied, the fine structure of EELS Ti-$L_{2,3}$ edge is changed in such a way that the t_{2g} peak becomes suppressed compared to the e_g peak as fewer core electrons are excited to t_{2g} and the Ti-$L_{2,3}$ edge shifts to lower energy (redshift) as the binding energy is lowered. We quantified the Ti^{3+} fraction by using multiple linear least square (MLLS) fitting of the EELS Ti-$L_{2,3}$ edge with the assumption that it consists of a linear combination of the signals from the Ti^{4+} and Ti^{3+} state. The periodic fluctuation in the Ti^{3+} fraction is clearly captured when the measured Ti^{3+} fraction is plotted with the distance from the interface (Fig. 3d). However, it should be mentioned that the measured Ti^{3+} profile can be spread out from the real profile because the EELS signal can also be generated from neighboring atomic sites by beam spreading and channeling.

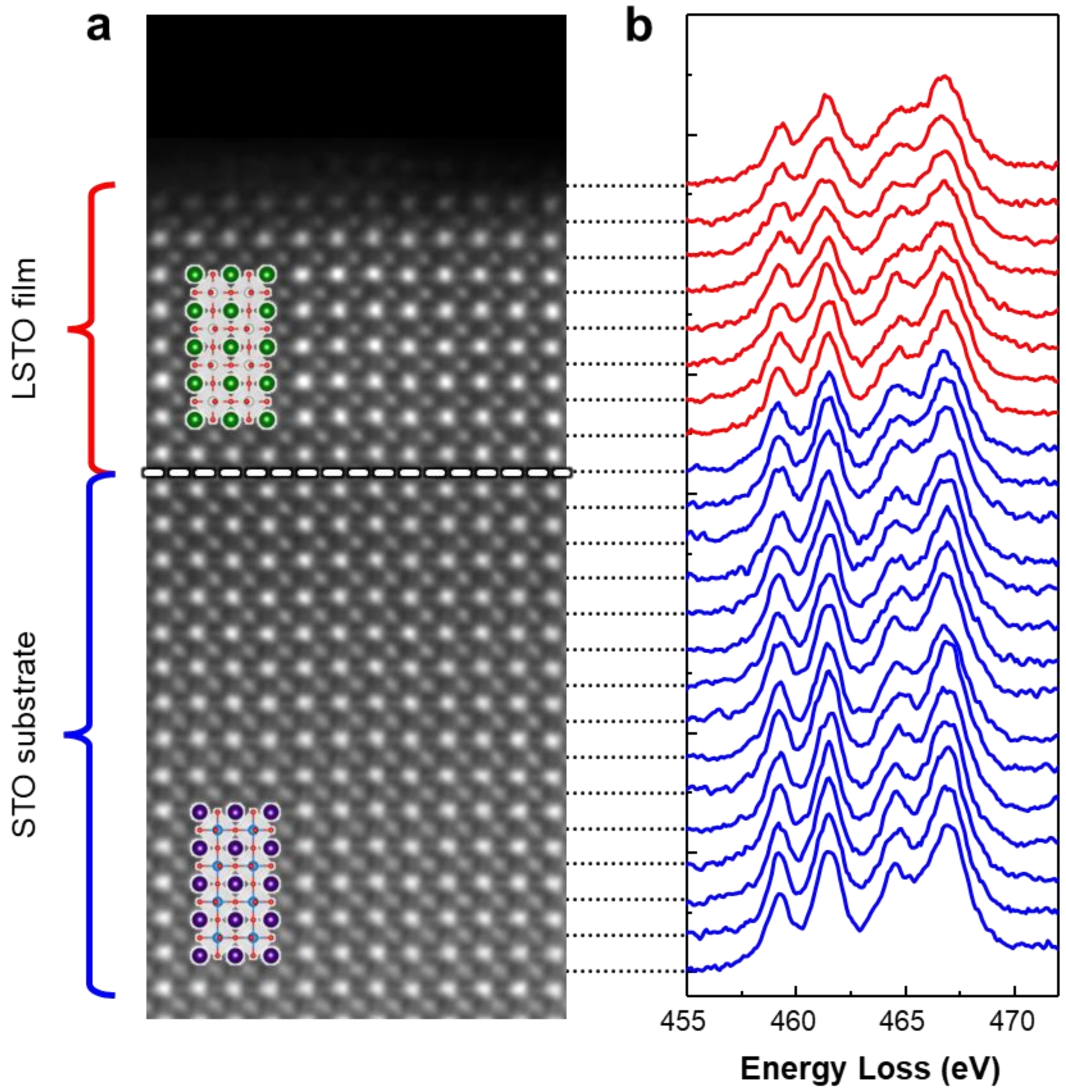

Supplementary Fig. 11 | Electron Energy Loss Spectra (EELS) of LSTO 8 u.c./STO heterostructure. a STEM images of the LSTO heterostructure**. b** Ti-$L_{2,3}$ edge spectra extracted from their line profiles.

Supplementary Note 8: DFT calculation and symmetry analysis of the LSTO/STO heterogeneous slab

As mentioned in the main article, we investigate LSTO/STO heterogeneous slab structure with various LSTO film thicknesses using the density functional theory (DFT) with semi-empirical Hubbard *U* correction like LSTO bulk. To describe the STO substrate, we first investigate the atomic and electronic structure of STO bulk. Considering the experimentally observed structure with the anti-phase octahedral rotation distortion ($a^0a^0c^-$ in Glazer's notation)[1,10] in STO, we optimize the lattice constants and the internal atomic coordination. The optimized in-plane and out-of-plane lattice constants are a = 0.39587 and c = 0.39866 nm, respectively. Based on the experimental observation of the LSTO films' $a^0a^0c^-$ Glazer's notation pattern as well as the STO substrate[1,2], we constructed the candidate structures of the LSTO/STO heterogeneous slab by La atom substituting a Sr atom site of the STO slabs.

Using the obtained atomic structure of STO bulk, we constructed a TiO_2-terminated STO mirror symmetric slabs with various thicknesses (including 8.5, 10.5, 12.5, and 14.5 unit cell layer) to investigate LSTO film on the STO substrate with varying film thickness. La atoms were substituted in one of four Sr atom sites on the SrO layers to construct LSTO film which corresponds to 25% La-doping concentrations. While 6 bottom SrO sublayers were not substituted to simulate 6.5 u.c. of the undoped TiO_2-terminated STO for the substrates, the LSTO films with various thickness, t (t = 2, 4, 6, and 8 u.c.), were placed on the top of the STO substrate (Supplementary Fig. 12). A vacuum, length of $4c$ ($4c$ = 1.59464 nm), is included in the slab structures, and the in-plane directions, $2a \times 2a$ (2a = 0.79174 nm) cell is used. Where a and c are fixed to those of the optimized value of the STO bulk. The atomic structures of the constructed LSTO/STO slab are optimized by relaxing the atomic positions with the atoms on the 3 bottom layers of the substrate fixed. When we performed the calculation using slab

structure, the dipole correction was used in the **z**-direction to alleviate the unintended external electric field resulting from the image supercell.

We also investigate the magnetic and electronic structures of the LSTO/STO slab. We consider several different magnetic configurations, and the total energy difference between different magnetic configurations is up to 6.5 meV. We find an *A*-type-like antiferromagnetic (AFM) spin alignment as the ground-state magnetic structure of the LSTO thin films for all t investigated, where Ti^{3+} has parallel spin alignment in the in-plane direction, and anti-parallel spin alignment in the out-of-plane direction (Fig. 4d, Supplementary Fig. 16b, Supplementary Fig. 17c, f and Supplementary Fig. 18c). We would like to note that the obtained magnetic state of the LSTO film, the *A*-type-like AFM order, is different from those of other rare-earth titanates with $a^-a^-c^+$ rotation pattern which has *G*-type AFM or ferromagnetic (FM)[4] order ($a^-a^-c^+$ Glazer's notation). The *A*-type-like AFM order can be stabilized by the $a^0a^0c^-$ rotation by super-exchange interaction[11,12]. We also calculate the electronic band structure and the density of states of the LSTO/STO slab as shown in Supplementary Figure 13 and Figure 4a. The slab is insulating when $t \leq 6$ u.c., while metallic when $t = 8$ u.c..

We analyze the lattice modulation of the LSTO film similar to the bulk LSTO. The lattice modulations of the LSTO films are decomposed into distortion modes, i.e., $\Delta r^t = \sum_i c^t_i \Phi^t_i$, where Δr^t is the lattice modulation vector of the LSTO film with the thickness t (t = 2, 4, 6, and 8 u.c.) defined as the atomic displacement vector from the symmetrical structure, c^t_i is the coefficients of the normalized distortion mode Φ^t_i (i = 1, 2, 3, z- and rem) with Φ^t_1: the inter-layer breathing mode, Φ^t_2: the intra-layer breathing mode, and Φ^t_3: the Jahn-Teller type distortion mode, Φ^t_{z-}: the antiferrodistortive rotation (Supplementary Fig. 14). For t = 8 u.c., $\Delta r^{8u.c.}$ and $\Phi^{8u.c}_i$ are defined for only the modulated LSTO (top 6 layers, 0th to -5th layers), i.e., the atomic displacements of the -6th and -7th layers are excluded in the definition of $\Delta r^{8u.c.}$ and

$\Phi^{8u.c}{}_i$. $d^t{}_i$s are oxygen displacements with respect to the high symmetric structures, which are defined as the multiply $c^t{}_i$s by individual oxygen displacements of the normalized distortion mode vectors $\Phi^t{}_i$s (i = 1, 2, 3, z-). Oxygen octahedral rotation angles $\theta^t{}_{z}$-s of antiferrodistortive rotation are converted from $d^t{}_{z}$-s. The obtained coefficients $c^t{}_i$s and oxygen displacements $d^t{}_i$s and oxygen octahedral rotation angles $\theta^t{}_z$ are summarized in Supplementary Tables 1 and 2, respectively. Note that for all the invested thickness (t = 2, 4, 6, and 8 u.c.), the remaining term ($\Phi^t{}_{rem}$) mainly come from the atomic displacements on the surface layer (Supplementary Fig. 15), and the displacements of atoms near the La_{Sr} dopants (Supplementary Fig. 3). These surface atomic displacements result in a downward atomic rumpling on the 0th TiO_2 sublayer (top surface) and an upward atomic rumpling on the -0.5^{th} (La, Sr)O layer. This DFT calculated rumpling behavior of the LSTO film surface is also reported in undoped TiO_2-terminated STO (001) surface (see Supplementary Note 9).

This slab structure also shows the periodic charge modulation of $Ti^{3+}O_6$ and $Ti^{4+}O_6$ with the expanded and contracted TiO_6 octahedra and exhibits the antiferromagnetic Mott insulating behavior with the localized d band when $t \leq 6$ u.c. (Fig. 4d, Supplementary Fig. 17c,f), indicating CO phase. Note that the obtained atomic and electronic structures of the LSTO/STO slab have similar characteristics to those of the CO phase in the bulk LSTO. Thus, we conclude that the metastable CO phase of bulk LSTO is stabilized in the LSTO/STO heterostructure (Fig. 4e).

When t = 8 u.c., the layer-resolved density of state (LDOS) shows the metallic state with the occupied conduction bands on the -8th and -9th layers, which is in the STO substrate (Supplementary Fig. 18). This indicates that the electrons originating from La_{Sr} dopants, which are located on the -6.5^{th} and -7.5^{th} sublayer, were transferred to the STO substrate due to the formation of LSTO/STO junctions[13]. The carrier spreading can evoke a metallic response from

a substrate even when the substrate was insulating. Since ionized impurity scattering center such as La_{Sr} dopant does not exist in STO substrate, these electrons can exhibit high mobility at low temperatures, resulting in an extremely large residual resistivity ratio (RRR) (Fig. 2d). It has been reported that low temperature electron mobility of LSTO thin films increased with the decrease in La-doping concentration, leading to a large RRR[14]. Therefore, we attribute that the large RRR of the 8 u.c. thick LSTO thin film originates from substrate conduction.

We would like to note that the calculated electronic structure of each layer might not perfectly match the experimental situation, since the La_{Sr} dopants in the calculation are regularly distributed unlike in the real sample case. However, the underlying physics of the surface-induced OSMT, the stabilization of the CO phase, and subsequent MIT are irrelevant to the doping distribution.

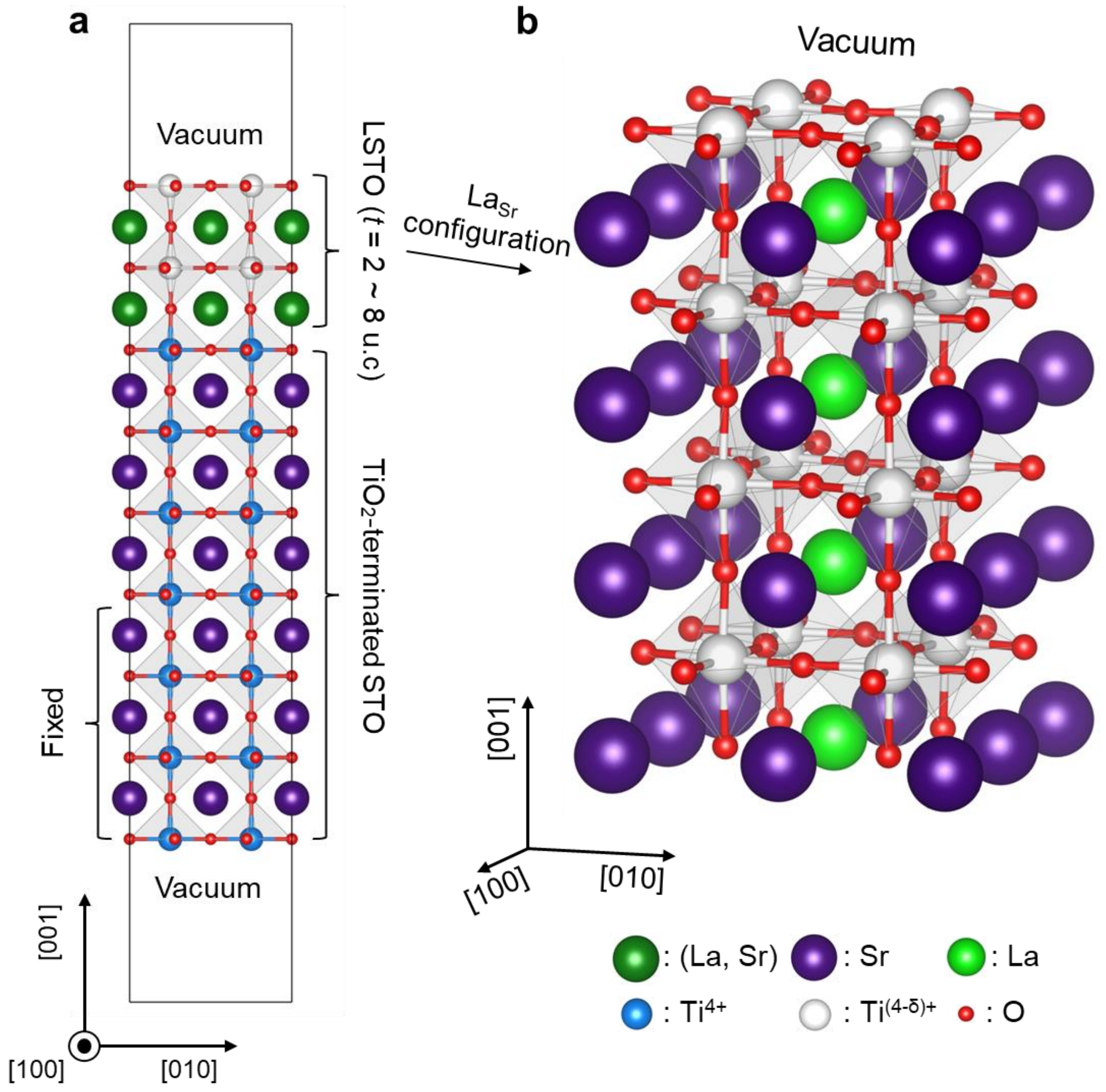

Supplementary Fig. 12 | Atomic structure of the LSTO/STO heterogeneous slab in DFT. **a** Schematic of the LSTO/STO heterogeneous slab structure used in the DFT calculation. The 6.5 layers of TiO_2-terminated STO substrate and the LSTO film with various thickness, *t* (*t* = 2, 4, 6, and 8 u.c.), are included in the heterogeneous slab. **b** Schematic of the La_{Sr} doping configuration in the LSTO film. La_{Sr} dopants are linearly aligned in the surface normal direction, i.e., [001] direction. Green, purple, chartreuse, red, blue, and white spheres represent the (La, Sr), Sr, La, O atoms, Ti^{4+} cation, and partially filled $Ti^{(4-\delta)+}$ cation, respectively.

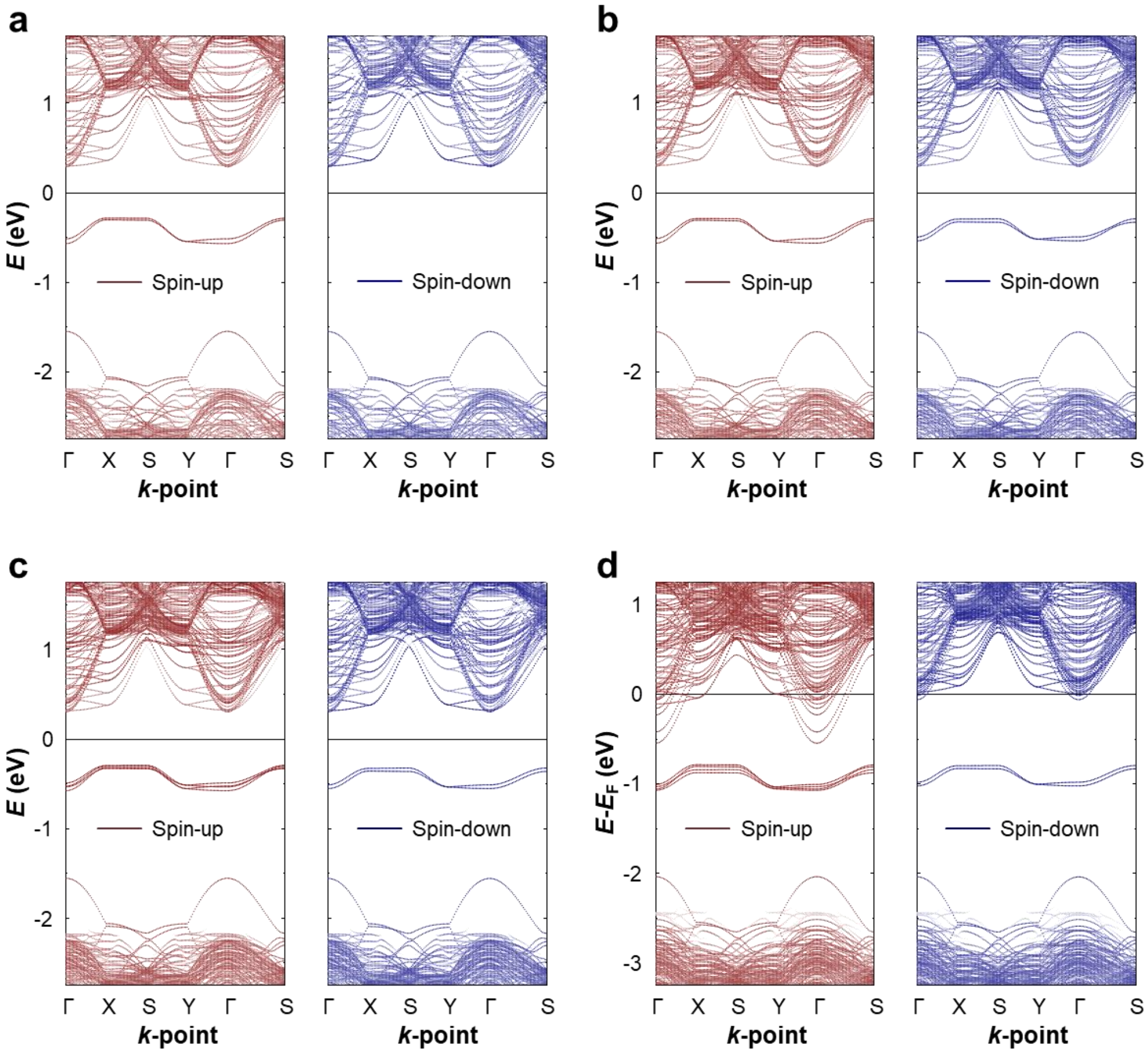

Supplementary Fig. 13 | DFT calculated spin-polarized band structure of the LSTO/STO slab. Spin-polarized band structures of the LSTO films on STO with various film thickness t = 2, 4, 6 and 8 u.c.. **a** 2 u.c., **b** 4 u.c., **c** 6 u.c. and **d** 8 u.c.. The bands are projected to minimize spurious electronic states originating from the fixed bottom surface layer of the slab, where the STO 2 u.c. from the bottom surface are excluded from the projection. Burgundy and indigo colors represent the spin-up and spin-down bands, respectively. For t = 2, 4, 6 u.c., the midpoint of the energies of the lowest unoccupied state and the highest occupied state is set to be zero and represented by the horizontal lines. For t = 8 u.c., the Fermi energy is set to be zero and represented by the horizontal lines.

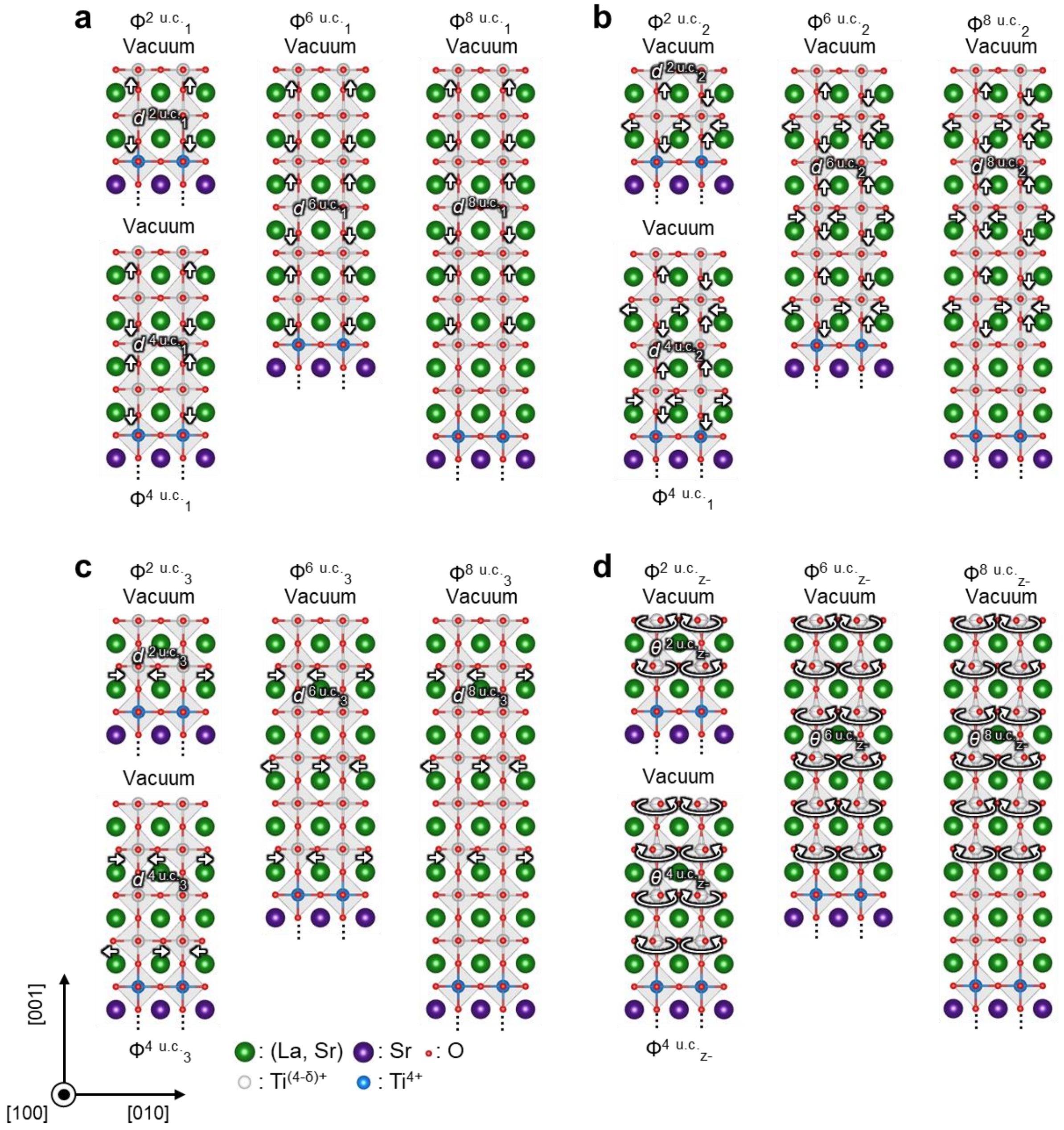

Supplementary Fig. 14 | Distortion modes for the LSTO film. The normalized distortion mode Φ^{t}_{i} are schematically shown (i = 1, 2, 3, and z-). **a** The inter-layer TiO_6 breathing mode Φ^{t}_{1}, **b** the intra-layer TiO_6 breathing mode Φ^{t}_{2}, **c** the Jahn-Teller type distortion Φ^{t}_{3}, **d** the antiferrodistortive rotation Φ^{t}_{z-}. Green, purple, red, white, and blue spheres represent the (La, Sr), Sr, and O atoms and $Ti^{(4-\delta)+}$, and Ti^{4+} cation, respectively.

Supplementary Table 1 | Analyzed coefficients of the normalized distortion modes of the LSTO films with various film thickness *t* = 2, 4, 6, and 8 u.c..

	c^t_1	c^t_2	c^t_3	c^t_{z-}	c^t_{rem}
t = 2 u.c.	0.0902	0.125	0.0720	0.753	0.828
t = 4 u.c.	0.136	0.178	0.0844	1.43	0.949
t = 6 u.c.	0.180	0.224	0.122	1.91	1.02
t = 8 u.c.	0.184	0.225	0.122	1.90	1.08

Supplementary Table 2 | Analyzed oxygen displacements and oxygen rotation angle of the LSTO films by the distortion modes with various film thickness *t* = 2, 4, 6, and 8 u.c..

	d^t_1 (pm)	d^t_2 (pm)	d^t_3 (pm)	θ^t_{z-} (°)
t = 2 u.c.	3.19	3.12	2.55	9.79
t = 4 u.c.	3.41	3.14	2.11	9.39
t = 6 u.c.	3.67	3.23	2.48	9.31
t = 8 u.c.	3.75	3.25	2.49	9.26

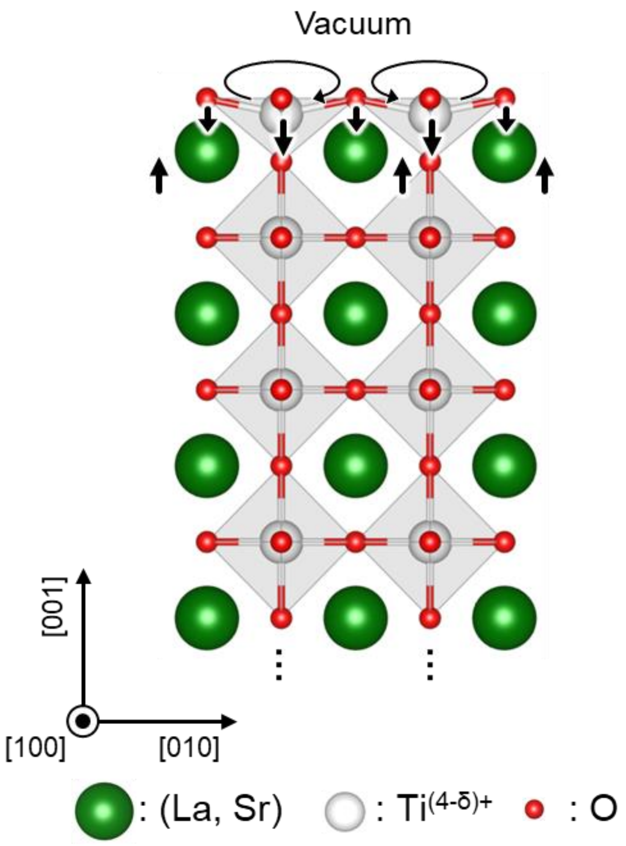

Supplementary Fig. 15 | Schematics of the remaining term Φ^{t}_{rem} for the LSTO/STO slab. For all the invested film thickness t (t = 2, 4, 6, and 8 u.c.), Φ^{t}_{rem} mainly consists of the displacements on the surface layer, and the displacements near La_{Sr} (Supplementary Fig. 3). Green, white, and red spheres represent the (La, Sr) atoms, $Ti^{(4-\delta)+}$ cation, and O atoms, respectively.

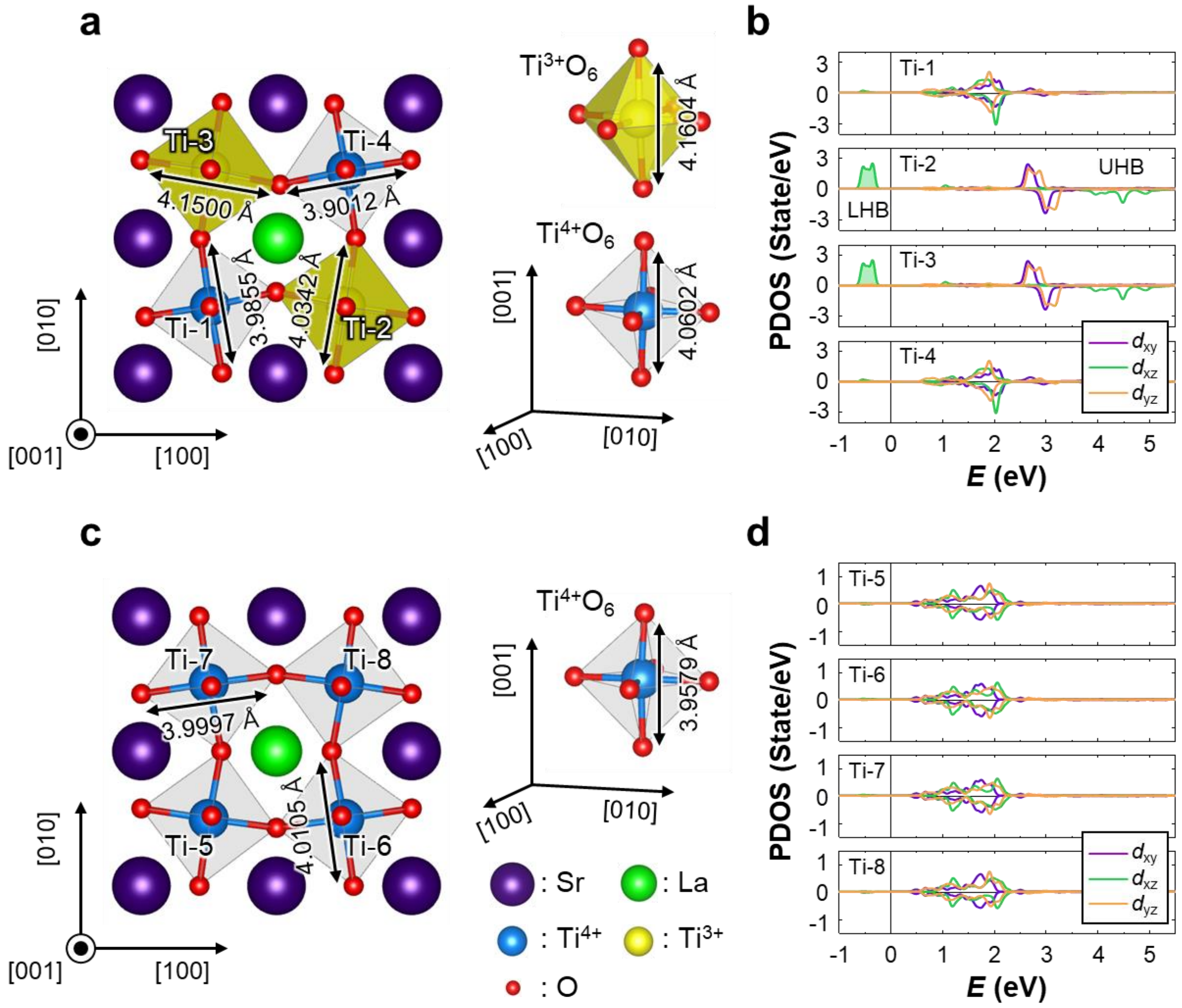

Supplementary Fig. 16 | Atomic and electronic structures of the LSTO film on STO substrate with *t* = 6 u.c.. **a** Atomic structure of the -1st layer of the film. **b** Spin-polarized density of states (DOS) projected onto the t_{2g} orbitals of Ti atoms on the -1st layer. **c** Atomic structure of -2nd layer of the film. **d** Spin-polarized DOS projected onto the t_{2g} orbitals of Ti atoms on the -2nd layer. In **a**, **c** purple, chartreuse, red, blue, and yellow spheres represent the Sr, La, and O atoms and Ti^{4+}, and Ti^{3+} cations, respectively. In **a** the yellow shades on the $Ti^{3+}O_6$ octahedral surface are guides for eyes. In **b**, **d** purple, chartreuse, and orange lines represent the DOS projected onto the Ti d_{xy}, d_{xz}, and d_{yz} orbitals, respectively. Positive and negative regions correspond to the up-spin and the down-spin states, respectively. The midpoint of the energies of the lowest unoccupied state and the highest occupied state is set to be zero and represented by the vertical line.

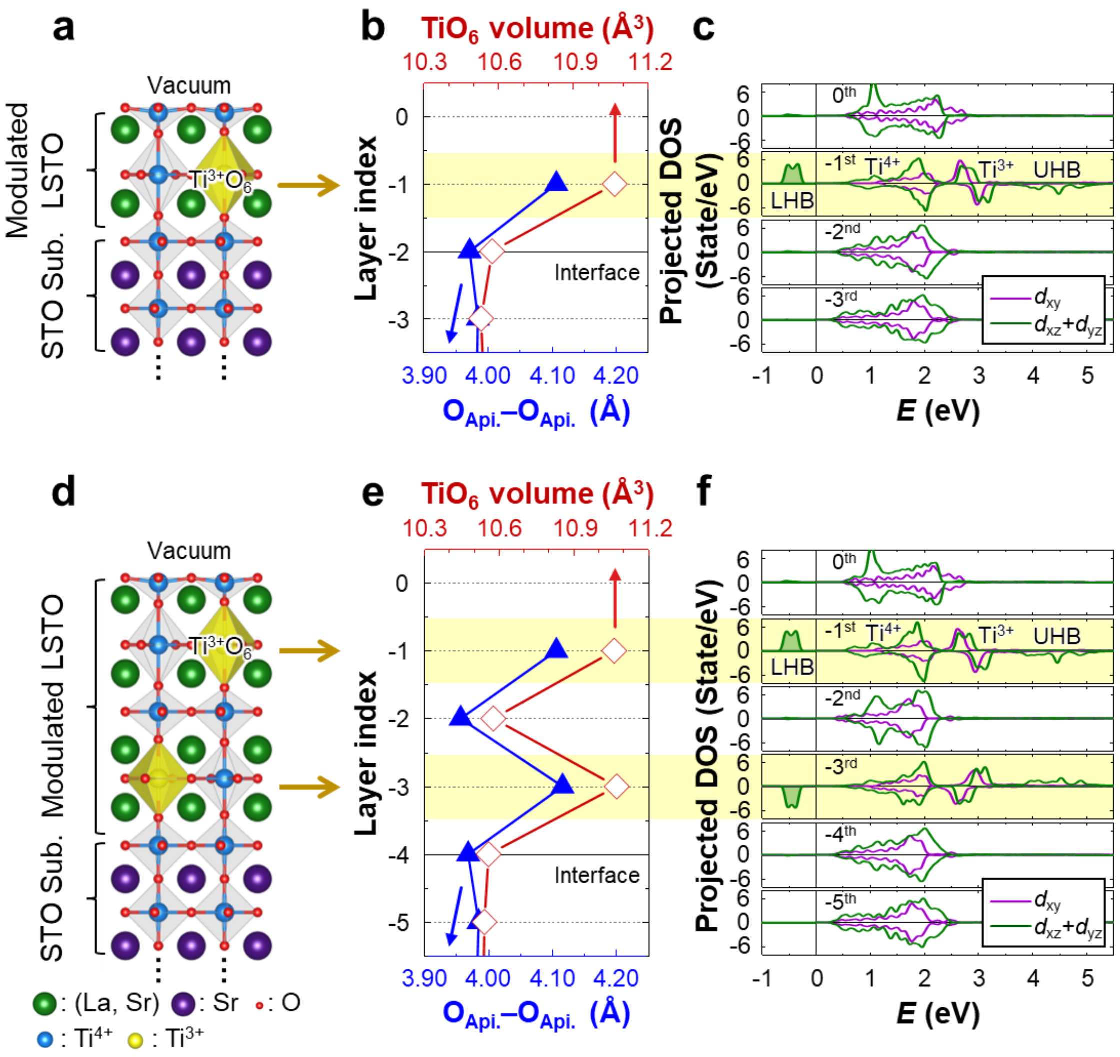

Supplementary Fig. 17 | Atomic and electronic structures of the LSTO/STO slab with *t* = 2 and 4 u.c.. a Schematic representation of the atomic structure of the LSTO film on STO (001) surface for thickness, $t = 2$ u.c.. **b** The height and volume of TiO_6 octahedra of the LSTO/STO heterostructures. The layer-averaged height and volume of the TiO_6 octahedra obtained from the DFT calculations are plotted. **c** The layer-resolved DOS projected onto the Ti $3d$ t_{2g} orbitals for $t = 2$ u.c.. **d** Schematic representation of the atomic structure of the LSTO film on STO (001) surface for $t = 4$ u.c.. **e** The height and volume of TiO_6 octahedra of the LSTO/STO heterostructures. The layer-averaged height and volume of the TiO_6 octahedra obtained from the DFT calculations are plotted. **f** The layer-resolved DOS projected onto the Ti $3d$ t_{2g} orbitals for $t = 4$ u.c.. In **a**, **d** green, purple, red, blue, and yellow spheres represent the (La, Sr), Sr, O atoms and Ti^{4+}, and Ti^{3+} cations, respectively. The yellow shades on the $Ti^{3+}O_6$ octahedral surface are guides for eyes. In **b**, **c** the light-gold highlights for the -1st sublayers are guides for eyes. In **b**, **e** the blue line with the closed triangles and the red line with the open diamonds represent the layer-averaged height and volume of TiO_6 octahedra, respectively. In **c**, **f** purple and green lines represent the DOS projected onto the Ti d_{xy} and $d_{xz}+d_{yz}$, respectively. Positive and negative regions correspond to the up-spin and the down-spin states, respectively. The midpoint of the energies of the lowest unoccupied state and the highest occupied state is set to be zero and represented by the vertical line. In **e**, **f** the yellow highlights for the -1st and -3rd sublayers are guides for eyes.

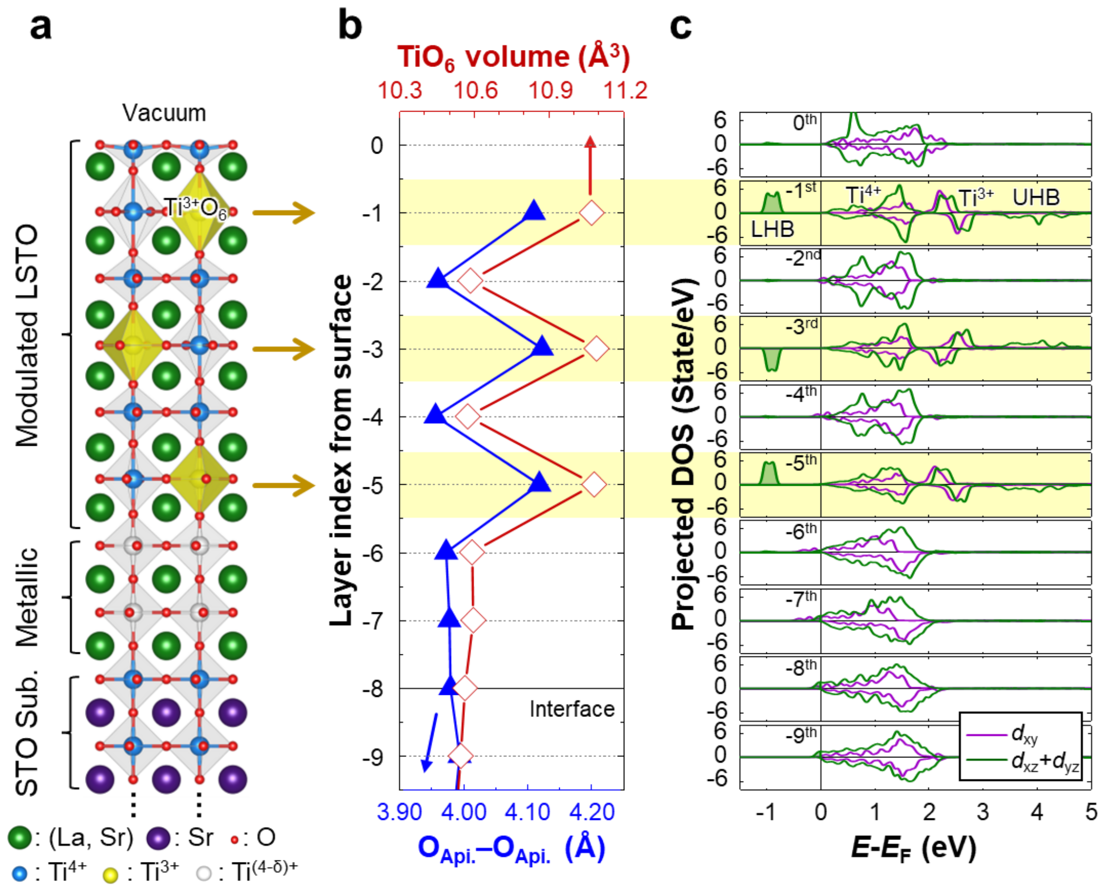

Supplementary Fig. 18 | Atomic and electronic structures of the LSTO/STO slab with t = 8 u.c.. a Schematic representation of the atomic structure of the LSTO film on STO (001) surface for thickness, t = 8 u.c.. Green, purple, red, blue, yellow, and white spheres represent the (La, Sr), Sr, O atoms and Ti^{4+}, Ti^{3+}, and partially filled $Ti^{(4-\delta)+}$ cations, respectively. The yellow shades on the $Ti^{3+}O_6$ octahedral surface are guides for eyes. **b** The height and volume of TiO_6 octahedra of the LSTO/STO heterostructures. The layer-averaged height and volume of the TiO_6 octahedra obtained from the DFT calculations are plotted. The blue line with the closed triangles and the red line with the open diamonds represent the layer-averaged height and volume of TiO_6 octahedra, respectively. **c** The layer-resolved DOS projected onto the Ti $3d$ t_{2g} orbitals for t = 8 u.c.. Purple and green lines represent the DOS projected onto the Ti d_{xy} and d_{xz}+d_{yz}, respectively. Positive and negative regions correspond to the up-spin and the down-spin states, respectively. The Fermi energy is set to be zero and represented by the vertical line. In **b**, **c** the yellow highlights for the -1st, -3rd, and -5th sublayers are guides for eyes.

Supplementary Note 9: Verification of surface structure

First, it is important to clarify whether surface reconstruction contributes to the emergence of the charge-ordered phase in LSTO thin film. Depending on the thermodynamic condition including temperature and pressure, several phases of $SrTiO_3$ (001) surface have been reported such as (1×1), (2×1), (2×2), *c*(4×2), (4×4), *c*(4×4), *c*(6×2), (6×2), (√5×√5)-R26.6° and (√13×√13)-R33.7° structure, respectively[15]. Typically, a reconstructed surface on STO is obtained through thermal annealing under ultra-high vacuum at relatively higher temperatures. It should be noted that the LSTO thin film was grown at a temperature of 650 °C (923.15 K) and 10^{-5} Torr of oxygen/ozone mixture, which is a relatively lower temperature and higher oxidation condition than that required for surface reconstruction.

We further investigated the surface structure using reflection high-energy electron diffraction (RHEED) before and after LSTO film growth, to verify any possibility of the presence of an in-plane reconstruction phase on the surface. We monitored the RHEED pattern of the pre-growth STO (001) surface and the post-growth LSTO thin film surface with [100] beam incidences (Supplementary Fig. 19). No additional superstructure spot was observed before or after LSTO film growth. Thus, we conclude that the surface of our LSTO thin film remains in an unreconstructed state, which is also reported[16-17]. In addition, before the film growth, we performed short re-etching procedure using buffered-HF to maintain a TiO_2 termination up to growth temperature (i.e., 650 °C)[18].

Secondly, to determine the surface structure of LSTO film, we explored the detailed atomic structure and surface atomic distortion of ultrathin LSTO thin films by complementary density functional theory (DFT) calculations and Coherent Bragg rods analysis (COBRA) studies (Supplementary Fig. 20). The surface distortion is usually initiated by the termination of a crystalline material, i.e., dangling bonds at the topmost surface. According to Pauling's

rule[19,20], an equilibrium cation-anion bond length strongly depends on the coordination number. Due to the decreased coordination number of the Ti and O atoms (TiO_6 octahedra to TiO_5 pyramid and Sr_4Ti_2O octahedra to Sr_2Ti_2O polyhedra) on the TiO_2 terminated $SrTiO_3$ surface, shortened Ti-O and Sr-O equilibrium bond lengths result in downward and upward rumpling on the 0th TiO_2 sublayer (top surface) and -0.5^{th} SrO sublayer, respectively[21]. Note that these surface atomic rumpling in the TiO_2 terminated LSTO film is well described in our DFT calculations (Supplementary Fig. 20).

To confirm this atomic rumpling behavior experimentally, we carried out synchrotron surface X-ray diffraction measurements and performed COBRA analysis of an LSTO thin film (t = 8 u.c.). The complete atomic structures of each unit cell of the LSTO thin film including the surface unit cell in focus can be obtained from COBRA analysis. Supplementary Figure 20 shows the COBRA derived full electron density map with 2D vertical slices on the (110) atomic plane and (200) atomic plane, respectively. The slices along the (200) atomic plane cuts through the Ti, apical oxygen, and equatorial oxygen sites. Therefore, we can quantitatively determine the Ti-O atomic rumpling ($\delta_{Ti\text{-}O}$) magnitude. Likewise, we identify (La, Sr)-O atomic rumpling ($\delta_{(La,\ Sr)\text{-}O}$) magnitude using the slice along the (110) atomic plane cutting through the (La, Sr), and apical oxygen sites. The slice along the (200) and (110) atomic planes reveal a downward rumpling on the 0th TiO_2 sublayer (top surface) and upward rumpling on the -0.5^{th} (La, Sr)O sublayer. Therefore, we conclude that experimentally determined surface structures such as atomic rumpling behavior at the surface layer are in good agreement with those obtained from the DFT calculations.

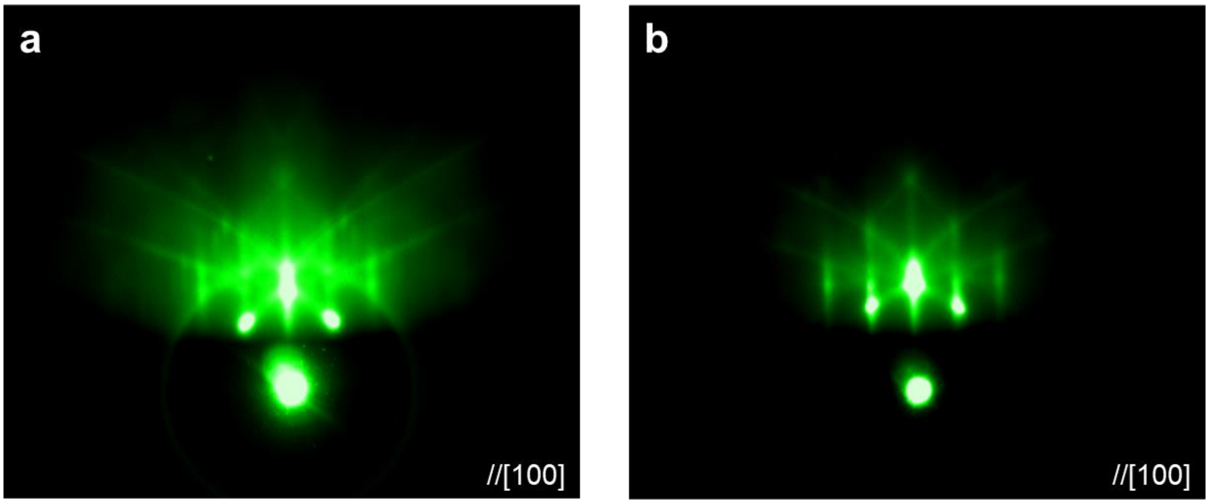

Supplementary Fig. 19 | RHEED patterns for LSTO thin film growth obtained at room temperature along the [100] azimuths. a RHEED pattern of the STO (001) surface after the annealing process. The STO substrate was etched 45 sec using buffered-HF and annealed at 1100 °C for 6 hours. Before the film growth, the second chemical etching procedure using buffered-HF for 10 sec was performed to stabilize the TiO_2-terminated surface of the STO substrates[18]. **b** RHEED pattern of the LSTO thin film surface after growth. The LSTO thin film is grown under 10^{-5} Torr of oxygen/ozone mixture and at 650 °C.

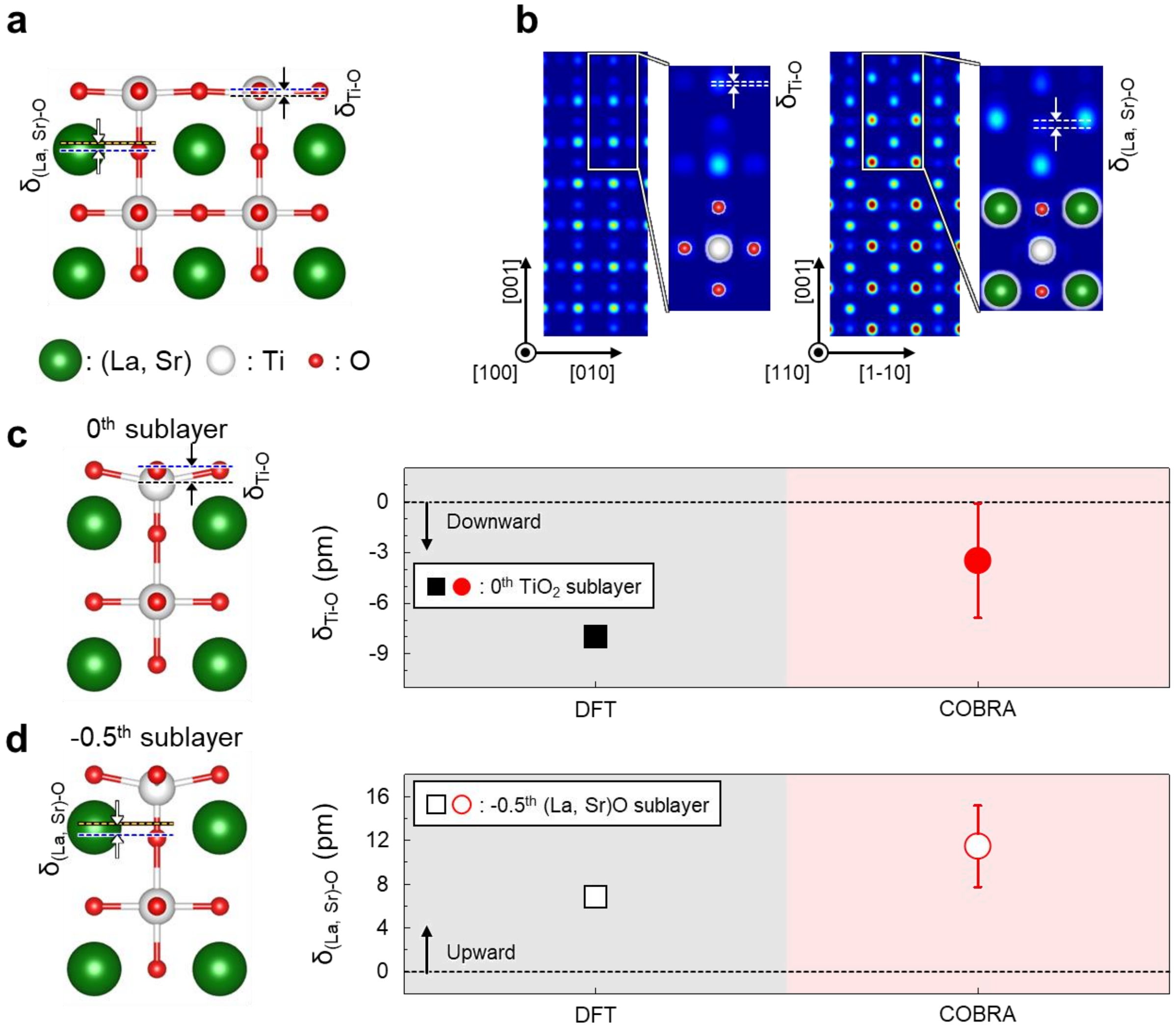

Supplementary Fig. 20 | a Calculated atomic structure near the surface of the 8 u.c. thick LSTO film on STO substrate. **b** A 2D vertical slice through the 3D electron density map along the (200) and (110) atomic plane for an 8 u.c. thick LSTO film. (La, Sr) and equatorial oxygen is not visible along the (200) and (110) atomic plane, respectively. **c-d** Atomic rumpling of the relaxed (100) surfaces, as a determination method: DFT and COBRA. Ti-O atomic rumpling ($\delta_{Ti\text{-}O}$) on the 0th sublayer (c) and (La, Sr)-O atomic rumpling ($\delta_{(La,\ Sr)\text{-}O}$) on the -0.5^{th} sublayer (d).

Supplementary Note 10: Effect of oxygen adsorption on the film surface for electrical properties

It is known that oxygen adsorbs on the film surface in ambient air and influence the electronic structure and consequent transport properties of the transition metal oxide. When the surface of metallic film is decorated with oxygen adsorbates, the occupation of *d* orbital can be decreased. This implies that the oxygen adsorbates drain electrons from the film surface, leading to the formation of a space charge region near the top surface region. However, it has been known that this effect is only confined to the surface regime, typically within 1 or 2 u.c. from the surface[22]. Considering relatively thicker critical thickness for MIT in ultrathin LSTO film than the thickness of an electrical dead layer originated from oxygen adsorbates[22], we believe that oxygen adsorption only plays a minor role in the electrical properties of the La-doped STO system.

Supplementary Note 11: Attenuation of the surface charge-ordered phase

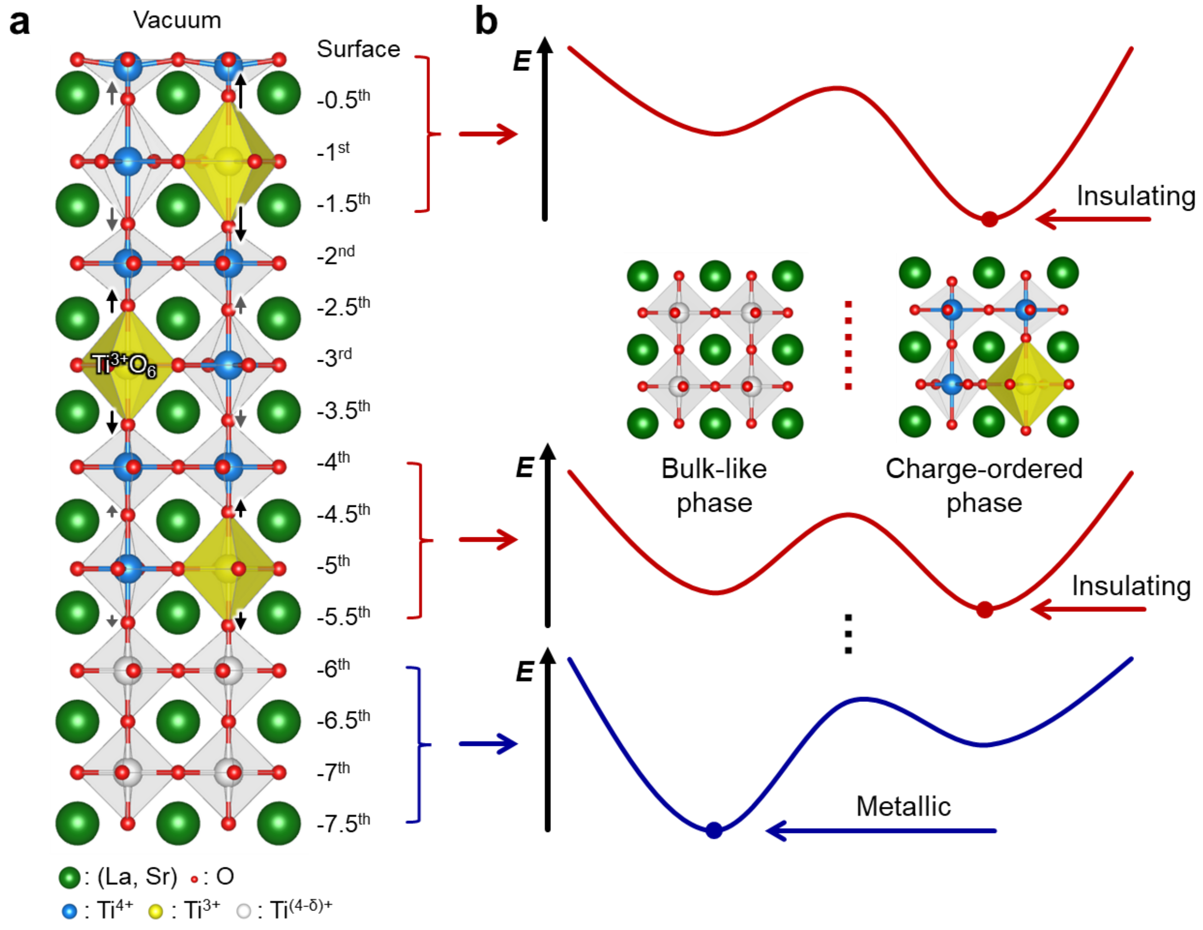

Supplementary Fig. 21 | Schematics of how the surface dangling bond triggers the charge-ordered instability in the surface region. **a** Schematic of apical oxygen displacement due to the surface dangling bond. A TiO_5 pyramid, formed at the top surface leads to a shortened equilibrium Ti-O bond length. The apical oxygen in the -0.5th sublayer was pushed upward which results in an elongated Ti-O bond length in the -1st layer. Therefore, Ti^{3+} states with the charge-ordered patten are stabilized in this layer, giving rise to an increase in the TiO_6 volume and pushing down on the apical oxygen in the -1.5th sublayer. Note that the apical oxygen raising in the -0.5th sublayer corresponds to the inter-layer breathing mode (Φ_1) (Fig. 4e). **b** Schematic of the energy landscape between bulk-like metallic phase and charge-ordered insulating phase depending on the depth, i.e., sublayer index. In the surface region, the charge-ordered insulating phase is stabilized by the surface dangling bond and this phase is attenuated at depth. In **a**, **b** green, red, blue, yellow, and white spheres represent the (La, Sr), O atoms and Ti^{4+}, Ti^{3+}, and partially filled $Ti^{(4-\delta)+}$ cations, respectively. The yellow shades on the $Ti^{3+}O_6$ octahedral surface are guides for eyes.

Supplementary Note 12: Charge-ordered phase in different La-doping concentration

To investigate how the doping concentrations affect the charge-ordered phase $La_xSr_{1-x}TiO_3$ system, we additionally investigate 15% La-doped STO thin film. Supplementary Figure 22 shows the thickness dependence of the carrier densities at the 15% and 25% La-doped STO thin films. The sheet carrier density (n_s) versus thickness for both La-doping concentrations shows a linear relationship and each linear fit intersects to $n_s = 0$ at nonzero thickness (Supplementary Fig. 22a). Below these thicknesses, the resistivity exceeds the measurement limit, indicating thickness-driven metal-to-insulator transition (MIT). The temperature-dependent resistivity of the 15% La-doped film with varying thicknesses also shows consistent results, implying thickness-driven MIT (Supplementary Fig. 22b).

To understand the underlying physics of the thickness-driven MIT at different doping concentrations such as 15% La_{Sr}, we investigated the atomic and electronic structures of the 12.5% La-doped STO film with 6 u.c. thickness on the STO substrate by first-principles calculations. To simulate atomic and electronic structures of 15% La-doped STO film on top of STO (001) using a conventional supercell approach, a supercell size should be increased by a minimum of $2\sqrt{2}a \times 5\sqrt{2}a$ along the in-plane direction, which has 20 Sr sites on every SrO sublayer. Considering computational resources, such a supercell approach is limited so calculation convergences cannot be achieved. Therefore, we first consider using virtual crystal approximation (VCA)[23] to simulate 15% La-doped STO film. Using VCA, we construct 15% La-doped STO film by replacing the La and Sr atoms' pseudopotentials with a weighted pseudopotential by La/Sr ratio 0.15/0.85, thus the size of the supercell can be effectively reduced. However, when the local potentials of the two atoms are not sufficiently comparable, the result of VCA is usually unreliable[24]. Hence, we decided to construct the 12.5% La-doped STO film on the STO substrate, which slightly deviates from 15% La_{Sr}, using the supercell

approach. In the in-plane directions, the $2\sqrt{2}a \times 2\sqrt{2}a$ cell, which has eight Sr sites on every SrO sublayer, is used to simulate 12.5% La-doped STO.

The calculation results show an insulating phase with the periodic modulation of the lattice and the electron density, i.e., alternating $Ti^{3+/4+}O_6$ and $Ti^{4+}O_6$ octahedral layers. Supplementary Figure 23 shows the obtained DFT results of the 12.5% La-doped STO film with 6 u.c. thickness on STO. The lattice modulation with $Ti^{3+/4+}O_6$ and $Ti^{4+}O_6$ octahedra is represented by schematics (Supplementary Fig. 23a). The layer-averaged height of TiO_6 octahedron (i.e., $O_{Api.} – O_{Api}$. distance), and the layer-averaged volume of TiO_6 (Supplementary Fig. 23b) are quantitatively analyzed, showing a periodic modulation of TiO_6 octahedron. The calculated layer-resolved density of states (DOS) shows the periodic modulation of the electron density (Supplementary Fig. 23c).

Similar to the charge-ordered phase in the 25% La-doped STO, TiO_6 octahedra expand in the out-of-plane direction in the -1st, -3rd, and -5th layers (odd-numbered sublayers). Consequently, the out-of-plane t_{2g} orbitals (d_{xz}/d_{yz} orbitals) of Ti ions in the expanded octahedra become localized and energetically stable. In contrast, the -2nd and -4th layers' TiO_6 octahedra slightly contract in the out-of-plane direction, and the energy levels of the t_{2g} states are relatively unchanged. As a result, all the electrons from the La_{Sr} dopants occupy the low-lying localized d_{xz}/d_{yz} band of the odd-numbered sublayers opening the Hubbard gap, while all the t_{2g} states of the even-numbered sublayers are unoccupied. Hence, the entire 12.5% La-doped STO film becomes an insulator that consists of alternating layers of Mott insulator (odd-numbered sublayers) and band insulator (even-numbered sublayers). This charge ordering (CO) along the out-of-plane direction is consistent with that observed in the 25% La-doped STO film (Fig. 4b-d). It should be noted that, as we described in the manuscript, expanded and contracted TiO_6 octahedra in the out-of-plane direction are originated from intra-layer breathing mode

(Φ_1), regardless of the doping concentration (Supplementary Fig. 24a).

On the other hand, we found that the CO along the in-plane direction strongly depends on La-doping concentration. In 25% La-doped STO, Ti^{3+}/Ti^{4+} ionic ordering has an in-plane checkerboard arrangement (Supplementary Fig. 24b), while, in 12.5% La-doped STO, Ti^{3+}/Ti^{4+} ionic ordering along the in-plane direction depends on the site configuration of La_{Sr}. By evaluating the stability of the several different configurations for the Ti^{3+}/Ti^{4+} ionic ordering arrangement along the in-plane direction, we found that the electrons originated from La_{Sr} tends to be localized at the nearest neighbor Ti sites of La_{Sr} (Supplementary Fig. 24c). This implies that the formation of diagonal rows of Ti^{3+}/Ti^{4+} ionic ordering is accompanied by La_{Sr}.

We expect that such an ordering of Ti^{3+}/Ti^{4+} with La_{Sr} is a primary building block for the charge-ordered phase in the La-doped STO system. The lattice periodicity along the [110] or the $[1\bar{1}0]$ direction for the ordering of Ti^{3+}/Ti^{4+} with La_{Sr} may depend on the La-doping concentration. In fact, similar types of charge-ordered stripes have been reported in commensurate[25] and incommensurate[26] carrier-doped manganite. The diagonal Mn^{3+} stripes are separated by the regions of Mn^{4+} ions and create a periodic array, that is charge-ordered phase[25]. The spacing between diagonal Mn^{3+} stripes is inversely proportional to the doping concentration. Therefore, we attribute that the CO phase is not limited to specific doping concentrations but can exist within a certain range of doping concentrations in the $La_xSr_{1-x}TiO_3$ system. We believe that these subjects will be an intriguing area for the future research.

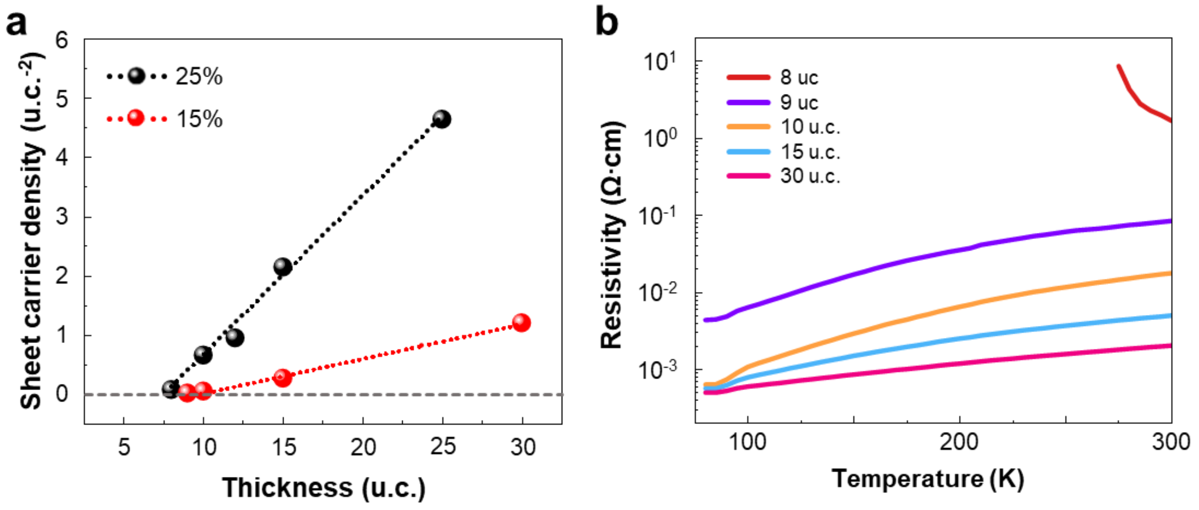

Supplementary Fig. 22 | a Sheet carrier density of 25% and 15% La-doped STO films as a function of the thickness measured at room temperature. The extrapolated dotted lines are linear fits to each plot. **b** Resistivity of 15% La-doped STO films with varying film thicknesses as a function of temperature range of 80 K to 300 K.

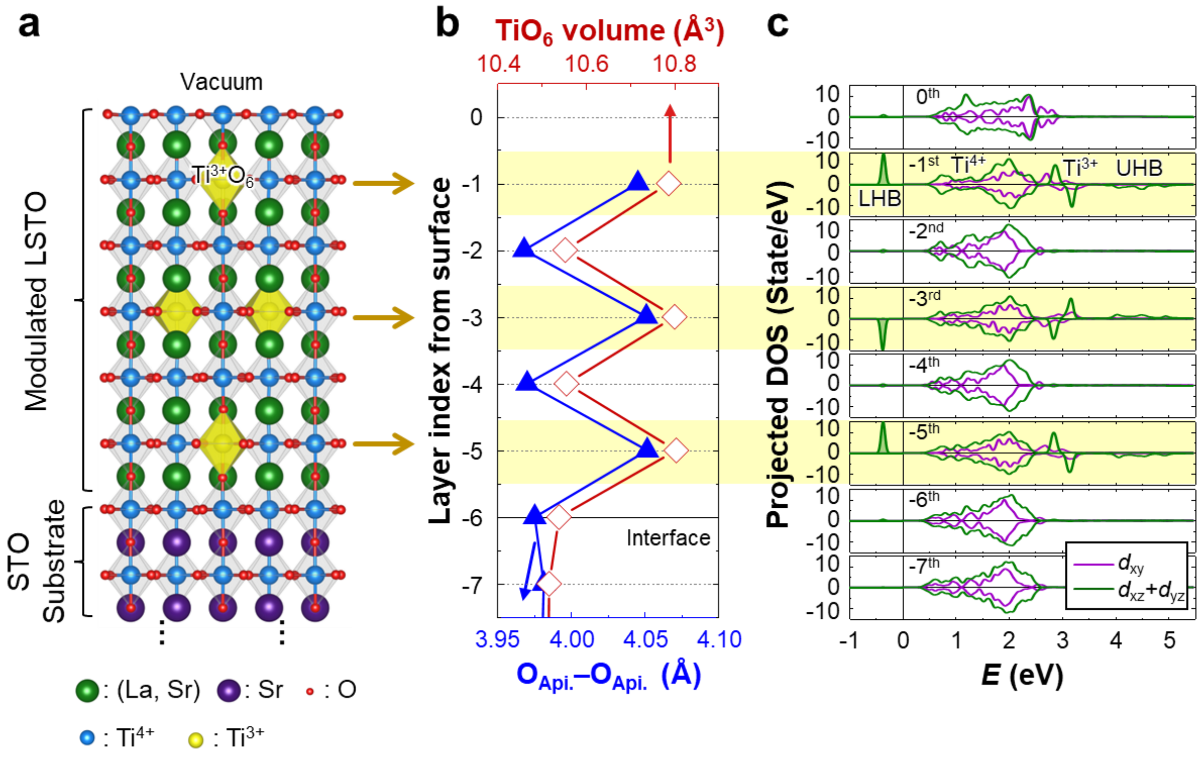

Supplementary Fig. 23 | Atomic and electronic structures of the 12.5% La-doped STO/STO slab with t = 6 u.c.. a Schematic representation of the atomic structure of the 12.5% La-doped STO film on STO (001) surface along [110] direction. Green, purple, red, blue, and yellow spheres represent the (La, Sr), Sr, O atoms and Ti^{4+}, and Ti^{3+} cations, respectively. The yellow shades on the $Ti^{3+}O_6$ octahedral surface are guides for eyes. **b** The height and volume of TiO_6 octahedra of the LSTO/STO heterostructures. The layer-averaged height and volume of the TiO_6 octahedra obtained from the DFT calculations are plotted. The blue line with the closed triangles and the red line with the open diamonds represent the layer-averaged height and volume of TiO_6 octahedra, respectively. **c** The layer-resolved DOS projected onto the Ti 3d t_{2g} orbitals. Purple and green lines represent the DOS projected onto the Ti d_{xy} and d_{xz}+d_{yz}, respectively. Positive and negative regions correspond to the up-spin and the down-spin states, respectively. The Fermi energy is set to be zero and represented by the vertical line. In **b**, **c** the yellow highlights for the -1st, -3rd, and -5th sublayers are guides for eyes.

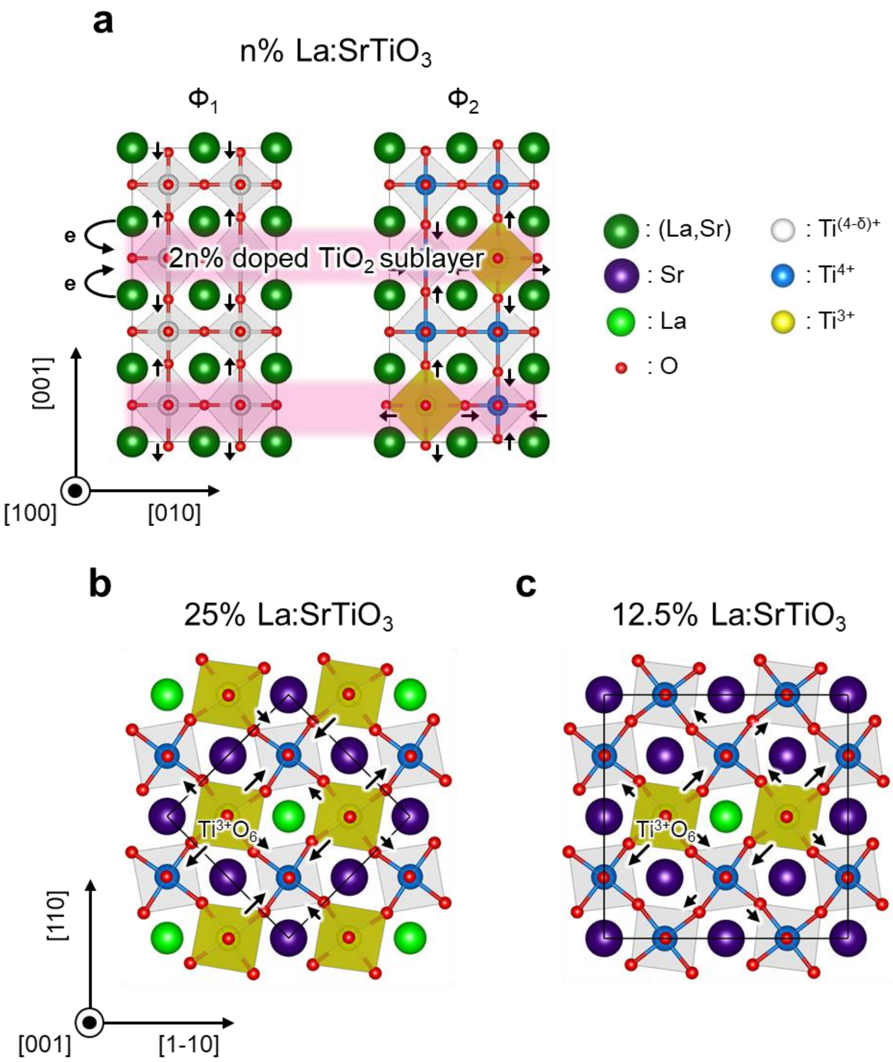

Supplementary Fig. 24 | Schematics of the Ti^{3+}/Ti^{4+} ionic ordering depending on various La-doping concentrations. a Charge ordering (CO) along the out-of-plane direction. Electrons are accumulated and localized due to the intra-layer breathing mode (Φ_1). **b**, **c** CO along the in-plane direction. The checkerboard arrangement of Ti^{3+}/Ti^{4+} ionic ordering of 25% La-doped STO (**b**). In 12.5% La-doped STO, diagonal rows of Ti^{3+}/Ti^{4+} ionic ordering accompanied by La_{Sr} are formed (**c**). In **a-c** green, purple, chartreuse, red, blue, yellow, and white spheres represent the (La, Sr), Sr, La, O atoms and Ti^{4+}, Ti^{3+}, and partially filled $Ti^{(4-\delta)+}$ cations, respectively. The yellow shades on the $Ti^{3+}O_6$ octahedral surface are guides for eyes.

Supplementary References

1. Sunstrom IV, J. E., Kauzlarich, S. M., & Klavins, P. Synthesis, structure, and properties of lanthanum strontium titanate $La_{1-x}Sr_xTiO_3$ ($0 \leq x \leq 1$). *Chem. Mater.* **4**, 346–353 (1992).

2. Shin., Y. J., Lau, C., Lee, S., Walker F. J. & Ahn, C. H. Surface-induced thickness limit of conducting La-doped $SrTiO_3$ thin film. *Appl. Phys. Lett.* **115**, 161601 (2019).

3. Bristowe, N. C., Varignon, J., Fontaine, D., Bousquet, E. & Ghosez, Ph. Ferromagnetism induced by entangled charge and orbital orderings in ferroelectric titanate perovskites. *Nat. Commun.* **6**, 6677 (2015).

4. Ouellette, D. G. et al. High-density two-dimensional small polaron gas in a delta-doped Mott insulator. *Sci. Rep.* **3**, 3284 (2013).

5. Mildner, S., Hoffmann, J., Blöchl, P. E., Techert, S. & Jooss C. Temperature- and doping-dependent optical absorption in the small-polaron system $Pr_{1-x}Ca_xMnO_3$. *Phys. Rev. B* **92**, 035145 (2015).

6. Bhargava, A. et al. Breakdown of the Small-Polaron Hopping Model in Higher-Order Spinels. *Adv. Mater.* **32**, 2004490 (2020).

7. Natanzon, Y., Amram, A. & Amouyal, Y. Evaluation of Polaron Transport in Solids from First-principles. *Isr. J. Chem.* **60**, 768–786 (2020).

8. Kim, S. J. et al. Optical Characterization of Doped $SrTiO_3$ in a Wide Photon Energy Region. *J. Korean. Phys. Soc.* **51**, 161–165 (2007).

9. Hoffmann, J. et al. Effects of interaction and disorder on polarons in colossal resistance manganite $Pr_{0.68}Ca_{0.32}MnO_3$ thin films. *Mater. Res. Express* **1**, 046403 (2014).

10. Unoki, H. & Sakudo, T. Synthesis, Electron Spin Resonance of Fe^{3+} in $SrTiO_3$ with Special Reference to the 110°K Phase Transition. *J. Phys. Soc. Jpn.* **23**, 546–552 (1967).

11. Solovyev, I. V. Superexchange interactions in orthorhombically distorted titanates RTiO$_3$ (R = Y, Gd, Sm and La). *New J. Phys.* **11**, 093003 (2009).

12. Fedorova, N. S. et al. Relationship between crystal structure and multiferroic orders in orthorhombic perovskite manganites. *Phys. Rev. Mater.* **2**, 104414 (2018).

13. Zhong, Z., & Hansmann, P. Band Alignment and Charge Transfer in Complex Oxide Interfaces. *Phys. Rev. X* **7**, 011023 (2017).

14. Son, J. et al. Epitaxial $SrTiO_3$ films with electron mobilities exceeding 30,000 $cm^2 V^{-1} s^{-1}$. *Nat. Mater.* **9**, 482–484 (2010).

15. Rocca, M., Rahman, T. S. & Vattuone, L. *Springer Handbook of Surface Science* 169–185 (Springer Press, 2020).

16. Naito, M. & Sato, H. Reflection high-energy electron diffraction study on the $SrTiO_3$ surface structure. *Physica C* **229**, 1–11 (1994).

17. Kajdos, A. P. & Stemmer, S. Surface reconstructions in molecular beam epitaxy of $SrTiO_3$. *Appl. Phys. Lett.* **105**, 191901 (2014).

18. Ohnishi, T. et al. Preparation of thermally stable TiO_2-terminated $SrTiO_3$(100) substrate surfaces. *Appl. Phys. Lett.* **85**, 272–274 (2004).

19. Pauling, L. THE PRINCIPLES DETERMINING THE STRUCTURE OF COMPLEX IONIC CRYSTALS. *J. Am. Chem. Soc.* **51** 1010–1026 (1929).

20. Pauling, L. *The nature of the chemical bond and the structure of molecules and crystals; an introduction to modern structural chemistry (3rd ed.)* 543–562 (Cornell University Press, 1960).

21. Saghayezhian, M., Rezaei Sani, S. M., Zhang, J. & Plummer, E. W. Rumpling and Enhanced Covalency at the $SrTiO_3$(001) Surface. *J. Phys. Chem. C* **123**, 8086−8091 (2019).

22. Gabel, J., Pickem, M. et al. Toward Functionalized Ultrathin Oxide Films: The Impact of Surface Apical Oxygen. *Adv. Electron. Mater.* **8**, 2101006 (2022).

23. Bellaiche, L. & Vanderbilt, D. Virtual crystal approximation revisited: Application to dielectric and piezoelectric properties of perovskites. *Phys. Rev. B* **61**, 7877 (2000).

24. Eckhardt, C., Hummer, K. & Kresse, G. Indirect-to-direct gap transition in strained and unstrained $Sn_xGe_{1−x}$ alloys. *Phys. Rev. B* **89**, 165201 (2014).

25. Mori, S., Chen, C. & Cheong, S.-W. Pairing of charge-ordered stripes in (La,Ca)MnO_3. *Nature* **392**, 473–476 (1998).

26. El Baggari, I. et al. Nature and evolution of incommensurate charge order in manganites visualized with cryogenic scanning transmission electron microscopy. *Proc. Natl. Acad. Sci. U.S.A.* **115**, 1445–1450 (2018).
